# Genomic co-localization of variation affecting agronomic and human gut microbiome traits in a meta-analysis of diverse sorghum

**DOI:** 10.1093/g3journal/jkae145

**Published:** 2024-07-09

**Authors:** Nate Korth, Qinnan Yang, Mallory J Van Haute, Michael C Tross, Bo Peng, Nikee Shrestha, Mackenzie Zwiener-Malcom, Ravi V Mural, James C Schnable, Andrew K Benson

**Affiliations:** Nebraska Food for Health Center, University of Nebraska-Lincoln, Lincoln, NE 68588, USA; Complex Biosystems Graduate Program, University of Nebraska-Lincoln, Lincoln, NE 68588, USA; Department of Food Science and Technology, University of Nebraska-Lincoln, Lincoln, NE 68588, USA; Nebraska Food for Health Center, University of Nebraska-Lincoln, Lincoln, NE 68588, USA; Department of Food Science and Technology, University of Nebraska-Lincoln, Lincoln, NE 68588, USA; Nebraska Food for Health Center, University of Nebraska-Lincoln, Lincoln, NE 68588, USA; Department of Food Science and Technology, University of Nebraska-Lincoln, Lincoln, NE 68588, USA; Complex Biosystems Graduate Program, University of Nebraska-Lincoln, Lincoln, NE 68588, USA; Center for Plant Science Innovation, University of Nebraska-Lincoln, Lincoln, NE 68588, USA; Nebraska Food for Health Center, University of Nebraska-Lincoln, Lincoln, NE 68588, USA; Department of Food Science and Technology, University of Nebraska-Lincoln, Lincoln, NE 68588, USA; Complex Biosystems Graduate Program, University of Nebraska-Lincoln, Lincoln, NE 68588, USA; Center for Plant Science Innovation, University of Nebraska-Lincoln, Lincoln, NE 68588, USA; Center for Plant Science Innovation, University of Nebraska-Lincoln, Lincoln, NE 68588, USA; Department of Agronomy and Horticulture, University of Nebraska-Lincoln, Lincoln, NE 68588, USA; Center for Plant Science Innovation, University of Nebraska-Lincoln, Lincoln, NE 68588, USA; Department of Agronomy and Horticulture, University of Nebraska-Lincoln, Lincoln, NE 68588, USA; Department of Agronomy, Horticulture, and Plant Science, South Dakota State University, Brookings, SD 57007, USA; Nebraska Food for Health Center, University of Nebraska-Lincoln, Lincoln, NE 68588, USA; Center for Plant Science Innovation, University of Nebraska-Lincoln, Lincoln, NE 68588, USA; Department of Agronomy and Horticulture, University of Nebraska-Lincoln, Lincoln, NE 68588, USA; Nebraska Food for Health Center, University of Nebraska-Lincoln, Lincoln, NE 68588, USA; Department of Food Science and Technology, University of Nebraska-Lincoln, Lincoln, NE 68588, USA

**Keywords:** microbiome, sorghum, genomics, gut microbiome, GWAS, in vitro fermentation, association study, nutrition in agriculture, health agriculture nexus, quantitative genetics

## Abstract

Substantial functional metabolic diversity exists within species of cultivated grain crops that directly or indirectly provide more than half of all calories consumed by humans around the globe. While such diversity is the molecular currency used for improving agronomic traits, diversity is poorly characterized for its effects on human nutrition and utilization by gut microbes. Moreover, we know little about agronomic traits’ potential tradeoffs and pleiotropic effects on human nutritional traits. Here, we applied a quantitative genetics approach using a meta-analysis and parallel genome-wide association studies of *Sorghum bicolor* traits describing changes in the composition and function of human gut microbe communities, and any of 200 sorghum seed and agronomic traits across a diverse sorghum population to identify associated genetic variants. A total of 15 multiple-effect loci (MEL) were initially found where different alleles in the sorghum genome produced changes in seed that affected the abundance of multiple bacterial taxa across 2 human microbiomes in automated in vitro fermentations. Next, parallel genome-wide studies conducted for seed, biochemical, and agronomic traits in the same population identified significant associations within the boundaries of 13/15 MEL for microbiome traits. In several instances, the colocalization of variation affecting gut microbiome and agronomic traits provided hypotheses for causal mechanisms through which variation could affect both agronomic traits and human gut microbes. This work demonstrates that genetic factors affecting agronomic traits in sorghum seed can also drive significant effects on human gut microbes, particularly bacterial taxa considered beneficial. Understanding these pleiotropic relationships will inform future strategies for crop improvement toward yield, sustainability, and human health.

## Graphical Abstract

**Graphical Abstract:**
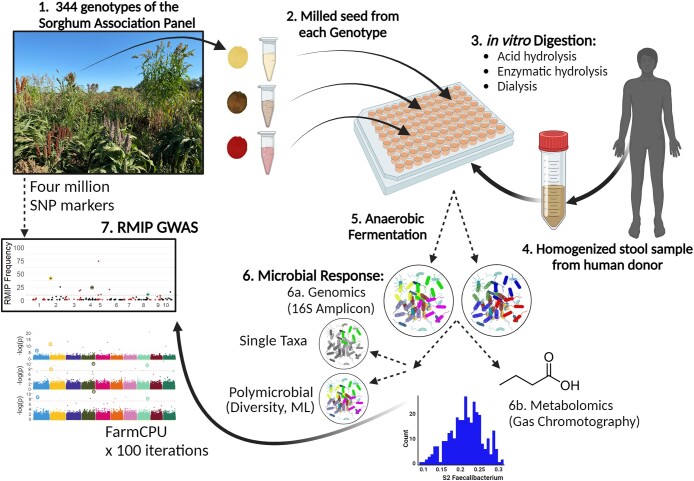
The Automated in vitro Microbiome screening (AiMs) platform. Varieties of the sorghum association panel, grown in Nebraska in 2020 were collected and ground into flour (1 and 2). Flour from each line was dispensed into 96-well plates and subjected to simulated human digestion, including acidification and enzymatic treatments that resemble digestion in the upper gastrointestinal tract. Small molecules (>1,000 KD) were removed by dialysis, simulating absorption in the small intestine (3). The digested material was fermented under anaerobic conditions with homogenized stool samples from human subjects (4 and 5). 16S amplicon sequencing and metabolomics were utilized to measure the microbial response to each sorghum genotype (6). All metrics describing the microbiome response (e.g. relative abundances of individual taxa and groups of taxa) were treated as a phenotype of sorghum in a Genome-Wide Association Study (GWAS) with a multiple resampling method implemented in Fixed and random model Circulating Probability Unification (FarmCPU) (7). The resampling model inclusion probability (RMIP)—the frequency a given marker is significantly associated with the phenotype in 100 GWAS tests using a random subset of the genotypes—of each marker was determined and plotted. This Diagram was created with BioRender.com.

## Introduction

The gut microbiome is a complex ecosystem of microorganisms residing in the gastrointestinal tract. Species composition and function of the gut microbiome have emerged as crucial determinants of both human health and predisposition to disease, contributing to the metabolism of nutrients, the synthesis of vitamins, the development of the immune system, the maintenance of the intestinal barrier, and the protection against pathogens. Dysbiosis, imbalances, or abnormal configurations in the composition and function of the gut microbiome has been linked to a number of health conditions in humans, including inflammatory bowel disease, diabetes, metabolic disorders, heart disease, and even mental health outcomes (Ley *et al.* 2005; Round *et al.* 2011; Le Chatelier *et al.* 2013; Halfvarson *et al.* 2017). While several factors shape species composition and function of the human gut microbiome, diet is one of the most significant determinants of both the abundance of different specific microbial taxa within the gut and overall human gut microbiome diversity (Ley *et al.* 2008; David *et al.* 2014). Different foods vary in the presence, absence, or content of dietary components that can each promote or inhibit the growth of specific bacterial taxa. The content and composition of digestion-resistant starches, fibers, and polyphenols found in food have been shown to have extensive impacts on gut microbes and human health in in vitro and in vivo systems (Maier *et al.* 2017; Jayarathne *et al.* 2019; Li *et al.* 2021).

Efforts to understand and harness the role diet plays in shaping the function and composition of the human gut microbiome have historically focused on different diets (e.g. Western vs Mediterranean) or have evaluated the effect of adding a known or suspected prebiotic compound, such as purified starches or fibers to an existing diet (Murga-Garrido *et al.* 2021; Wang *et al.* 2021; Kumar *et al.* 2022). Comparatively less effort has been made to investigate the relationships between naturally occurring genetic and metabolic/nutritional diversity within individual food crops and the potential impact of such diversity on the human gut microbiome. Extensive genetic and metabolite diversity exists within the primary gene pools of many of the most widely cultivated and consumed crops around the globe and amid such diversity, segregating genetic variants within crop species can control not only agronomic traits of the plant, but also the presence or relative abundance of bioactive compounds that can influence the composition of the human gut microbiome. In the case of sorghum (*Sorghum bicolor*), a grain closely related to maize that acts as a dietary staple and key component of food security in numerous parts of Africa and South Asia, substantial variation exists in the quantity and identity of complex fibers, resistant starch, and phenolic compounds—including condensed tannins (Elkhalifa *et al.* 2004; Noerhartati and Rahayuningsih 2016). Data from our recent in vitro microbiome fermentation studies with grain from functional genetic variants that affect starch composition (waxy) and seed protein composition (opaque) in individual varieties of sorghum and maize respectively have shown such variants can produce dramatic changes in the composition and function of human gut microbiomes (Korth *et al.* 2022; Yang *et al.* 2023).

In addition to functional genetics, we have also used quantitative genetics techniques to understand how plant genetic variation influences the human gut microbiome (Yang *et al.* 2022). This is a powerful strategy that employs Automated in vitro Microbiome Screening (AiMS) as a means for high-throughput in vitro phenotyping of the effects of hundreds of individual genotypes of a given crop species on the human gut microbiome. AiMS was developed because of the intractability of phenotyping large panels of genotypes in vivo through feeding studies. Such phenotyping would require the seed of each sorghum genotype to be fed sequentially to human volunteers, where individuals would be asked to consume the food made from one genotype for at least 1 week, followed by stool collection, several days washout, and consumption of seed from next genotype for 1 week + stool collection, etc. For a large panel such as the Sorghum Association Panel (SAP), such phenotyping would take > 10 years and other environmental effects on the microbiome occurring over the 10-year period would certainly confound the phenotyping. A number of in vitro digestion–microbiome fermentation strategies have been developed to overcome the limitations of human feeding studies. These include the complex, multicompartment SHIME and TIM-2 systems (Molly *et al.* 1993; Minekus *et al.* 1999) and continuous flow minibioreactors (Lattermann and Büchs 2015). Though simpler than feeding studies, none of these systems would afford the throughput for screening the hundreds to thousands of lines necessary for quantitative genetic analysis. Consequently, we focused on the more simplistic single-stage batch digestion–fermentation methods, and subsequently miniaturized and automated several steps in the digestion process to develop Automated in vitro Microbiome Screening (AiMS). The AiMS system was recently described and used in a proof-of-concept mapping study with a set of ∼300 sorghum recombinant inbred lines (RILs) developed from a biparental cross (Yang *et al.* 2022). This strategy uses a homogenized and filtered stool sample from an individual, which is dispersed in deep-well 96-well plates containing seed from each genotype of interest in individual wells that have been through in vitro digestion reactions. Variation in the microbiome fermentation patterns (as measured by 16S amplicon sequencing) can then be related to genetic variation across the sorghum genome by quantitative genetic analysis using methods such as quantitative trait locus (QTL) mapping and Genome-Wide Association Studies (GWAS).

In its initial implementation, the AiMS strategy allowed us to identify previously unknown genetic loci in sorghum-controlling seed components that cause quantitative changes in microbiome fermentation patterns. We also demonstrated how allelic effects at individual loci could be validated efficiently across microbiomes from multiple individuals, how candidate alleles could be identified, and ultimately how mechanistic studies could define how genetic variation affected seed components and how such variation affected specific gut microbes (Yang *et al.* 2022). The same study also identified several other loci where genetic variation in sorghum had substantial effects on the gut microbiome and could lead to the discovery of novel molecules and pathways through which genetic variation could affect desirable and undesirable gut microbes.

In the present study, we employed a sorghum diversity panel representative of global sorghum genetic and phenotypic diversity (Casa *et al.* 2008; Boatwright *et al.* 2022) and an AiMS microbiome phenotyping to identify genetic loci in sorghum associated with change in the composition or function of gut microbiomes from different human donors. In parallel to AiMS microbiome phenotyping, we also mapped loci that affect any of 200 different agronomic traits and observed significant associations of important agronomic traits within the multiple-effect loci (MEL) that also have substantial effects on microbiome composition. Novel sorghum traits and candidate genes from this study could be applied to breeding platforms that focus on crop nutrition and human health outcomes in sorghum and/or related species. Moreover, the overall strategy of AiMS phenotyping and GWAS can be applied to a wide range of diverse crops.

## Materials and methods

### Plant germplasm and growth conditions

The seed for the SAP (Casa *et al.* 2008) used in this study was grown in field conditions in 2020 at the University of Nebraska–Lincoln's Havelock farm (N° 40.861, W° 96.598). This field experiment has been previously described in detail (Grzybowski *et al.* 2022); briefly 344 sorghum genotypes listed in Supplementary Table 1 were planted on June 08, 2020. Each plot consisted of a single 1.5-m row of plants of a single genotype with 0.76-m spacing between parallel and sequential rows. Two to three panicles per plot were randomly selected and hand-harvested on October 16, 2020. Sorghum seed was dried and cleaned before being processed.

### Biochemical analysis of grain samples

The tannin content of each sorghum genotype was quantified using an established vanillin acidification protocol (Price *et al.* 1978). Briefly, 1 mL of 4% HCl in methanol was added to 25 mg of ground sorghum grain and incubated at 30°C for 20 min. Samples were centrifuged and a 100-µL aliquot was added to 500 µL 0.5% vanillin 4% HCl in methanol and a 4% HCl blank and incubated for 20 min. Absorbance at 500 nm was measured using a UV/VIS spectrophotometer. Final tannin contents were calculated by subtracting the absorbance of the blank from the absorbance of the sample and vanillin and comparing it to a catechin standard. Tannin content is reported as mg catechin equivalent lines. Lines that contained less than <1 mg catechin equivalent tannins were classified as “non-tannin” lines in downstream analysis.

Seed protein, oil, and starch concentrations were estimated using near-infrared reflectance (NIR). Each line was dried, cleaned, and then scanned using a Perten DA 7250 NIR spectrometer (Perten Instruments, Hägersten, Sweden). A random sample of the seed harvested from the selected panicles was scanned on the instrument using the small-sized sample tray. This measurement procedure was repeated 5 times per plot with different samples of grain (sampled with replacement). Scans that produced negative estimated values for any grain component were dropped. For each seed protein, oil, and starch, the median value across all remaining samples within a genotype/line was used for downstream analysis.

Data describing sorghum seed color was collected using a pretrained Mask R-CNN model on rice seeds to detect and segment the seeds from sorghum seed scans obtained using an EPSON scanner (Toda *et al.* 2020). Once the model performed pixel-level segmentation for the region of interest (ROI), seed regions in the scanned images, three-pixel values; red, green, and blue (RGB) channels were extracted for each ROI using custom Python code. For each color channel in a seed scan, the pixel values across all ROIs were averaged. This averaged RGB data as well as 3 principal components (PCs) calculated using the prcomp function in *R* was utilized for further analysis.

### Collection of publicly available SAP phenotypes

In addition to the newly generated sorghum phenotypes generated above, a set of 172 trait datasets mined from previously published studies of the SAP (Mural *et al.* 2021) were included in the GWAS to identify potential overlap between genes/genomic loci influencing directly observable sorghum traits and those influencing variation in the human gut microbiome (Supplementary Table 2).

### AiMs processing, sequencing, and short-chain fatty acid analysis

Fecal sample-derived microbiomes were collected from 12 volunteers; 8 fecal microbiomes were analyzed in the pilot screen, 2 in the mapping experiment, and all 12 in downstream validation. Stool samples were diluted in phosphate buffer saline with 10% glycerol and homogenized using an Interscience BagMixer 400 and filtered with Labplus 6 × 9 Filtra Bags. The filtrate was immediately aliquoted and frozen at −80°C for storage. The University of Nebraska Institutional Review Board (approval number 20160816311EP) approved the sample collection method. Approximately 3 g of sorghum seed for each of 344 lines was milled in a GenoGrinder 2025 (SPEX SamplePrep, Metuchen, NJ, USA) with 2, 7/16″ stainless steel ball bearings in 15-mL polycarbonate vials at 1,600 rpm for 6 min. Twenty milligrams (± 0.05 mg) of milled flour was dispensed into 96-well plates in a randomized complete block design with a Flex Powderdose GDU-p (Chemspeed Technologies AG, Füllinsdorf, Switzerland). Two different in vitro microbiome experiments were conducted: one for screening a small number of SAP across multiple microbiomes (initial screening study) and the second full-scale testing of the entire SAP across 2 selected microbiomes. Both experiments were laid out in a complete random block design with 3 technical replicates of each microbiome × sorghum genotype combination. For both experiments, a block consisted of four, 96-well plates—approximately 376 samples—with each line of sorghum present in at least 1 well in each block. In the initial screening experiment (microbiomes from 8 subjects × 24 sorghum genotypes × 3 technical replicates = 576 samples total), each block contained microbiomes from 4 human subjects and 24 different lines from the SAP. In the full-scale mapping experiment, each block contained the microbiome of a single human subject, and the 344 SAP genotypes were randomized across the four 96-well plates. Each block was repeated 3 times for each subject × genotype combination (1 microbiome × 344 genotypes × 3 technical replicates = 1,032 samples per subject not including blanks and controls). Sorghum flour was digested following established protocols (Yang *et al.* 2022). In brief, sorghum flour was hydrated in 425 µL water and cooked in boiling water for 20 min, agitating at 30-s intervals. Samples were treated with 45 µL of 500 mM HCl and 10% pepsin (P7000; Sigma, 470 St. Louis, MO, USA) at 37°C for 1 h. The small intestinal phase was initiated by adding sodium maleate buffer (pH = 6, containing 1 mM CaCl2) and NaHCO3. Pancreatin (P7545; Sigma, St. Louis, MO, USA) with amyloglucosidase (E-AMGDF, 473 3,260 U/mL, Megazyme) was added before incubation at 37°C for 6 h. Post digestion, samples were transferred to 96-well dialysis plates (molectular weight cut-off 1000; 475 DispoDialyzer; Harvard Apparatus, Holliston, MA, USA) and dialyzed in five gallons of distilled water replaced at 12-h intervals for 72 h at 4°C with agitation to facilitate the removal of small molecules. The digested and dialyzed sorghum flour was transferred to 1-mL 96-well plates. Fifty microliters of fecal microbiomes and 50 µL of 10 × fermentation media containing 1 g Bacto casitone, 1 g yeast extract, 2 g K2HPO4, 3.2 g NaHCO3, 3.5 g NaCl, 1 mL hemin solution (KOH 0.28 g, 95% Ethanol 25 mL, hemin 100 mg, and ddH2O to 100 mL), 0.05 g bile salts, 0.5 g/L cysteine HCl, 0.6 mL resazurin (0.1%), 10 mL ATCC trace mineral supplement, 3.6 mL volitile fatty acid solution (17 mL acetic acid, 1 mL n-valeric acid, 1 mL iso-valeric acid, 1 mL iso-butyric acid mixed with 20 mL of 10 mM NaOH), 10 mL ATCC vitamin supplement and 1 mL vitamin K-3 solution (0.14 g vitamin K-3 in 100 mL 95% ethanol) (Reichardt *et al.* 2018) reduced by the addition of 50% Oxyrase (SAE0013; Sigma, St. Louis, MO, USA) were added in an anaerobic chamber. Fermentations were incubated for 24 h at 37°C. Samples were centrifuged, bacterial pellets were used to determine microbiome composition, and the supernatant was retained for short-chain fatty acids (SCFA) analysis. Both pellets and supernatant were stored at −80°C until processing.

DNA was extracted from bacterial pellets in the BioSprint 96 workstation (Qiagen, Germantown, MD) and the BioSprint 96 one-for-all Vet Kit utilizing a stool lysis buffer (19082; Qiagen, Germantown, MD) and bead beating. Sequencing of the V4 region of the 16S rRNA gene was achieved by amplification and indexing by PCR primers as previously described (Caporaso *et al.* 2010). Libraries were normalized using a SequalPrep Normalization Plate (96) Kit (A1051001; Invitrogen, Frederick, MD). Paired-end sequencing was performed on an Illumina MiSeq (Kozich *et al.* 2013) at the Nebraska Food for Health Center. An average of 31,664 reads per sample was obtained.

Dada2 within the QIIME2 program (Callahan *et al.* 2016) was employed to extract amplicon sequence variants (ASVs) and assign each taxonomy based on the 132nd release of the SILVA 16S reference database (Quast *et al.* 2013). Forward and reverse reads were trimmed to 220 and 160 bp, respectively, to remove low-quality sequence data. ASVs present in only a single sample or composed of <15 reads total were removed from the analysis as were all microbial taxa at family, genus, and ASV levels with <5 reads in 75% of the samples. Read count was normalized by the cumulative sum squaring method with the Metagenomeseq (Paulson *et al.* 2013) package in R and converted to relative abundance for downstream analysis. Baseline microbiome samples and microbes fermented in media only (absent of sorghum) were collected as controls.

### Quantification of *Faecalibacterium prausnitzii*

The abundance of *F. prausnitzii* was measured using a quantitative PCR utilizing primers: FprauF: TGAGGAACCTGCCTCAAAGA; FprauR: GACGCGAGGCCATCTCA developed by Lindstad *et al.* (2021). The quantitative polymerase chain reaction (qPCR) reactions, conditions, and standard curve calculation were as previously reported (Yang *et al.* 2022).

### Variance partitioning analysis

The responses of gut microbiomes of eight human donors to a subset of 24 sorghum genotypes listed in Supplementary Table 1 were evaluated. For each ASV observed in at least 20% of samples, the variance was partitioned using the normalized relative abundance of each taxon with a linear mixed model using the relative abundance of a single genus of bacteria for a given subject as a response and fitting sorghum genotype, color, and tannin content as random effects in the Sommer package in R (Covarrubias-Pazaran 2016). Information on sorghum color and condensed tannin content was collected from publicly available data (Morris *et al.* 2013).

### Detection and removal of outliers among technical replicates

A 3-stage outlier detection and removal procedure was employed for microbiome traits prior to quantitative genetic analysis. In the first stage, the Jaccard index of beta diversity was calculated for each sample. For technical replicates, defined as samples generated using the same sorghum grain sample and treated with the same human gut microbiome, any single sample that was >0.2 from the midpoint of all technical replicates for that combination of sorghum genotype and human gut microbiome was removed. In the second stage, the mean and standard deviation for each microbial trait, including diversity, relative abundance, SCFA, and polymicrobial traits were calculated. Individual values >5 standard deviations from the mean were dropped, while other values from the same technical replicate were retained in the analysis. In the third stage: within replicates, an upper and lower threshold was set by calculating the upper and lower quantiles of the data and adding to the upper and subtracting from the lower, the interquartile range × 2.5, values outside this range were removed. Across all stages, less than 1% of the data was identified as outliers.

### Estimating the heritability of microbial traits

Narrow sense heritability (proportion of phenotypic variation due to genotypic variation) was estimated using a linear mixed model as implemented in the Sommer package in R (Covarrubias-Pazaran 2016). For each microbiome phenotype normalized, relative abundance data was input into a linear mixed model fit to block, plate number, and sorghum genotype identity as random effects. Reported heritability values are the quotients of variance in the response explained by the sorghum genotype divided by the sum of the variance in the response explained by the sorghum genotype and residual error as shown in the following equation:


$$h^{2}=\frac{\sigma_{g}^{2}}{\left(\sigma_{g}^{2}+\sigma_{r}^{2}\right)},$$

where $\sigma_{g}^{2}$ represents the variance in the microbial signature explained by genotype and $\sigma_{r}^{2}$ represents the variance explained by residual error.

### Best linear unbiased estimates calculation and estimates of polymicrobial traits

A linear mixed model was fit to each trait using the Sommer package (Covarrubias-Pazaran 2016) in *R* to calculate the best linear unbiased estimates (BLUEs) for microbiome signatures for each human subject's microbiome independently. Sorghum genotype was fit as a fixed effect. Block, plate, row, and column were fit as random effects as shown in the following equation:


$$Y_{ijklm}=G_{ij}+B_{i}+\;\gamma_{i}(P_{ij})+\delta_{ij}(R_{ijk})+\delta_{ij}(C_{ijk})+\varepsilon_{ijklm},$$

where $Y_{ijklm}$ represents the microbiome response variable for the *i*th Genotype in the *j*th Block, the *k*th plate within that block, and the *l*th row and *m*th column within that plate. $\gamma_{i}$ and $\delta_{ij}$ represent the coefficients associated with the interaction between block and plate and plate and row/column, respectively. $G_{ij}$ is the *i*th genotype in the *j*th block, $B_{i}$ is the *i*th block, $P_{ij}$ is the *i*th plate in the *j*th block, $R_{ijk}$ and $C_{ijk}$ are the *i*th row and column, respectively, of the *j*th plate and *k*th block, and $\varepsilon_{ijklm}$ represents the residual error. The equation was implemented in *R* using the Sommer function predict.mmer to calculate BLUEs for each microbial metric.

### Polymicrobial traits calculated from 16s rRNA sequencing data

One hundred PCs summarizing a matrix of the BLUE values for the 50 ASVs with the highest estimated heritabilities from each subject (100 ASVs in total) were calculated using the prcomp function in *R* with the center and scale parameters set to true generating 100 PC scores for each sorghum genotype.

Four estimated prebiotic potential index (PPI) values were calculated using the following equations. The first value was calculated based on the equation: PPIa, the sum of beneficial microbes minus detrimental bacteria, and is denoted as PreInd. The second value based on PPIb is transformed based on the difference of each bacterium incorporated into their mean value and is denoted as PreIndT. We recognize that identifying genera with detrimental impacts on human health solely from observational and associative data is difficult and frequently inconclusive. To mitigate the impact of errors in these identifications, we also calculated and employed indexes which only consider beneficial organisms denoted as PreIndB (nontransformed) and PreIndBT (transformed). Bacterial genera were determined as beneficial, detrimental, or inconclusive by searching the current literature for information about short-chain fatty acid production and association with known disease states. A list of organisms accounted for by the prebiotic index is reported in Supplementary Table 3.


$$\begin{array}{rl} \mathrm{PPIa}:\;\mathrm{PI}\;= & \frac{\mathrm{Reads}\;\;\mathrm{mapping}\;\;\mathrm{to}\;\;\mathrm{beneficial}\;\;\mathrm{bacteria}}{\mathrm{Total}\;\;\mathrm{Reads}}\\ & -\frac{\mathrm{Reads}\;\;\mathrm{mapping}\;\;\mathrm{to}\;\;\mathrm{detrimental}\;\;\mathrm{bacteria}}{\mathrm{Total}\;\;\mathrm{Reads}} \end{array}$$

$$\mathrm{PPIb}:\;\mathrm{PI}=\underset{\;p=1}{\sum }\;(x_{p}-\mu_{p})-\underset{n=1}{\sum }\;(x_{n}-\mu_{n})$$

where *x_p_* and *x_n_* represent the relative abundance of a given beneficial or detrimental microbe respectively and *µ* represents the mean value of that microbe. Prebiotic index correlated strongly with butyrate production in both human subjects (Supplementary Fig. 1).

An autoencoder neural network was employed to extract 10 latent variables describing sample-to-sample variation in patterns of microbial abundance. As separate sets of taxa were present in each subject, separate encoder and decoder architectures were trained on datasets from subject 1 and subject 2. Encoders and decoders were trained on the raw data (e.g. before consolidating technical replicates) after the detection and removal of outlier samples. The dataset for subject 1 comprised 1,073 samples described by 99 heritable microbiome traits. The dataset for subject 2 comprised 1,075 samples described by 106 heritable microbiome traits. Missing values for individual traits were imputed using the median value for the same trait among other samples that contained the same grain from the same sorghum genotype as the missing sample. In each case, the data was split at a ratio of 4:1 into training and validation sets.

The autoencoder model was implemented using PyTorch [v1.10.2 (Paszke *et al.* 2019)] package and the python (v3.9) programming language (Van Rossum *et al.* 2009). The model consisted of an encoder containing an input layer, three hidden layers, and an output layer as well as a decoder also containing an input layer, three hidden layers, and an output layer. For subject 1, the layers consisted of 99, 220, 300, 224, and 10 neurons in the encoder and 10, 224, 300, 220, and 99 neurons in the decoder. For subject 2, the layers consisted of 106, 220, 300, 224, and 10 neurons in the encoder and 10, 224, 300, 220, and 106 neurons in the decoder. The number of neurons in the first layer of the encoder and final layer of the decoder was set equal to the number of microbiome traits describing each subject. In the final layer of the decoder a hyperbolic tangent activation function was employed while for all other layers, the activation function used was the scaled exponential linear activation unit. The model was trained for 1,000 epochs using a mean absolute error loss function and a standard gradient descent optimization algorithm at a learning rate of 0.1. The best model was saved based on the lowest reconstruction loss on the validation dataset after each epoch. Final trained encoders were used to summarize the variance in each technical replicate. BLUEs for each of the 10 autoencoder-derived latent variables derived from each subject (20 total latent variables) were calculated as described above.

### Genome-wide association study

A marker set consisting of approximately 43 million genetic markers (e.g. SNPs, indels, and structural variants) derived from whole genome resequencing of the sorghum panel (Boatwright *et al.* 2022) was used in GWAS analysis. Sorghum accessions phenotyped in this study and not resequenced as part of Boatwright *et al*., were removed, reducing the number of genotypes to 340. Markers with a minor allele frequency of <0.1 and markers scored as heterozygous in more than 10% of sorghum lines genotyped were removed, resulting in a final set of 4,090,874 markers employed for GWAS. For each trait, including agronomic traits from Mural *et al*., and BLUEs from traits measured in this study (e.g. tannin concentration, seed composition, seed color, and microbial traits) GWAS was conducted using the Fixed and random model Circulating Probability Unification (FarmCPU) algorithm (Liu *et al.* 2016) as implemented in a memory-efficient, visualization-enhanced, and parallel-accelerated tool for Genome-Wide Association Study (rMVP) (Yin *et al.* 2021). For each trait, the first 3 PCs of genetic marker variation –calculated using rMVP—were included as covariates. Kinship was controlled as described in the FarmCPU algorithm. Resampling model inclusion probability (RMIP) was calculated by analyzing each trait 100 times, each with a random 10% of the data masked (Valdar *et al.* 2009). Markers were considered significant if they had a *P*-value <0.05/861,521.36, a threshold set by a Bonferroni correction using the effective number of markers calculated by the genetic type 1 error calculator, (Li *et al.* 2012). Genetic markers were considered significant if they were detected with a *P*-value lower than the significance threshold in at least 10 of the 100 FarmCPU iterations corresponding to an RMIP threshold of 0.1.

### Defining major effect loci

To identify regions potentially harboring allelic variants with major effects on the human gut microbiome, each chromosome was divided into 75 equally sized bins. As the sizes of each sorghum chromosome are distinct, this division method produced bin sizes ranging from 0.79 to 1.07 Mb for different chromosomes. A thresholding strategy was used to identify bins containing significant GWAS hits (defined as an RMIP value ≥10) for at least 5 microbial traits derived from each of the 2 human subjects. Hits for additional traits present in genomic bins adjacent to those selected for manual examination were merged as appropriate to control for the arbitrary placement of bin boundaries. Borders of bins meeting the threshold were then adjusted around the most frequent associated marker in the bin (marker affecting the most traits) using linkage disequilibrium (LD) of genetic markers within 100 kb of the most frequent significant using the TASSEL program (Bradbury *et al.* 2007). MEL regions were defined by the outer edges of the candidate gene space surrounding a given cluster of linked GWAS hits. All markers in high LD (*r*^2^ > 0.5) with significant markers were included in linkage blocks constituting final MEL regions. MEL were grouped based on their likelihood to correspond to a single causal variant, a small number of independent variants, or many variants within the MEL boundaries based on the LD of associated markers in the region defining the MEL and are denoted by linkage type: high, split, or low. LD among all markers identified as significantly linked to at least 1 microbiome trait within a given MEL was used to categorize MEL with high linkage (maximum *r*^2^ > 0.5 and the average *r*^2^ > 0.05), split linkage (maximum *r*^2^ > 0.5 and the average *r*^2^ < 0.05), or low linkage (maximum *r*^2^ < 0.5).

### Candidate gene detection

A list of genes within the boundary of MEL was compiled based on the *gff3* gene annotation file for the v3.1 build of the sorghum genome (Goodstein *et al.* 2012). If <5 genes were present within the boundaries of MEL, the window was expanded by 50 kb in both directions. Transcriptional data from sorghum 5 and 10 days after pollination, and embryo and endosperm 25 days after pollination were obtained from publicly available data (McCormick *et al.* 2018) and used to highlight genes that are expressed in the seed and filter out genes not expressed in the seed. Genes where all seed expression values measured in fragments per kilobase of transcript per million, were less than 1 were removed from further analysis.

### Validation of MEL6A haplotype

Sorghum genotypes were pooled into 4 categories based on tannin content and allele at the marker associated with the most microbial metrics in MEL6A (S06_43330339). Two pools of each of the 4 groups were made from 10 randomly selected sorghum genotypes, and 2.5 g of each pool was processed in the same digestion protocol described above. Fermentations were conducted in vitro using the fecal microbiomes of 12 human subjects collected as previously described on the sorghum pools with three technical replications. DNA extraction, 16S amplicon sequencing, and qPCR were conducted as described above.

### Statistical analysis

Data processing, compilation of diversity metrics, PERMANOVA, Wilcoxon tests, principal component analysis, BLUE calculation, FarmCPU GWAS, marker binning, and data visualization were conducted in the R statistical framework v4.0.2 (R Core Team 2020) utilizing the packages Sommer v4.1 (Covarrubias-Pazaran 2016), phyloseq v1.42.0 (McMurdie and Holmes 2013), Metagenomeseq v1.4 (Paulson *et al.* 2013), tidyverse v2.0 (Wickham *et al.* 2019), rMVP v1.0.6 (Yin *et al.* 2021), circlize (Gu *et al.* 2014), and ggplot2 v3.4.2 (Wickham 2009). BLASTp, hosted on the National Center for Biotechnology Information web service (States and Gish 1994; Sayers *et al.* 2022) was used to align protein sequences and calculate sequences similarly. Linear discriminant analysis effect size (LefSE) analysis was performed in the galaxy web application (Segata *et al.* 2011; Mandreoli *et al.* 2023). Gene functional annotations and analysis of gene sequence variation were based on the v3.1.1 sorghum reference database (McCormick *et al.* 2018) accessed via the Phytozome database (Goodstein *et al.* 2012). The InterPro database within Phytozome was used to assign functional annotations to candidate genes. R code to conduct the analyses described in this paper is available at https://github.com/natekorth/SAP.

## Results

### Variation among sorghum genotypes explains substantial variation in human gut microbiomes

Seed from 344 lines of the SAP grown as part of small plot field trials in Nebraska in 2020 (Grzybowski *et al.* 2022) was screened across the microbiomes of 2 human subjects.

In this study, the microbiomes of 2 human subjects were selected from a set of 8 candidate subjects. These 2 microbiomes were selected based on baseline composition and how microbial communities responded to in vitro fermentation with grain samples from a subset of 24 sorghum lines selected from the SAP. A variance partitioning analysis determined that the microbiomes of the 2 selected subjects (S766 and S777) included the largest number of microbial taxa where more than 20% of the total variance could be attributed to differences between sorghum genotypes (Supplementary Table 4). The 2 selected microbiomes are referred to as subject 1 (S766) and subject 2 (S777) below. Notably, tannin content in the sorghum lines explained much of the variation in the abundance of different microbial taxa present in subject 1 whereas the microbiome of subject 2 was substantially less tannin responsive, implying that the magnitude of responses of the 2 microbiomes to variation in the same seed component (e.g. tannins) was distinct, a condition which we expect by choosing microbiomes with unique species composition and unique fermentation patterns.

**Fig. 1.**
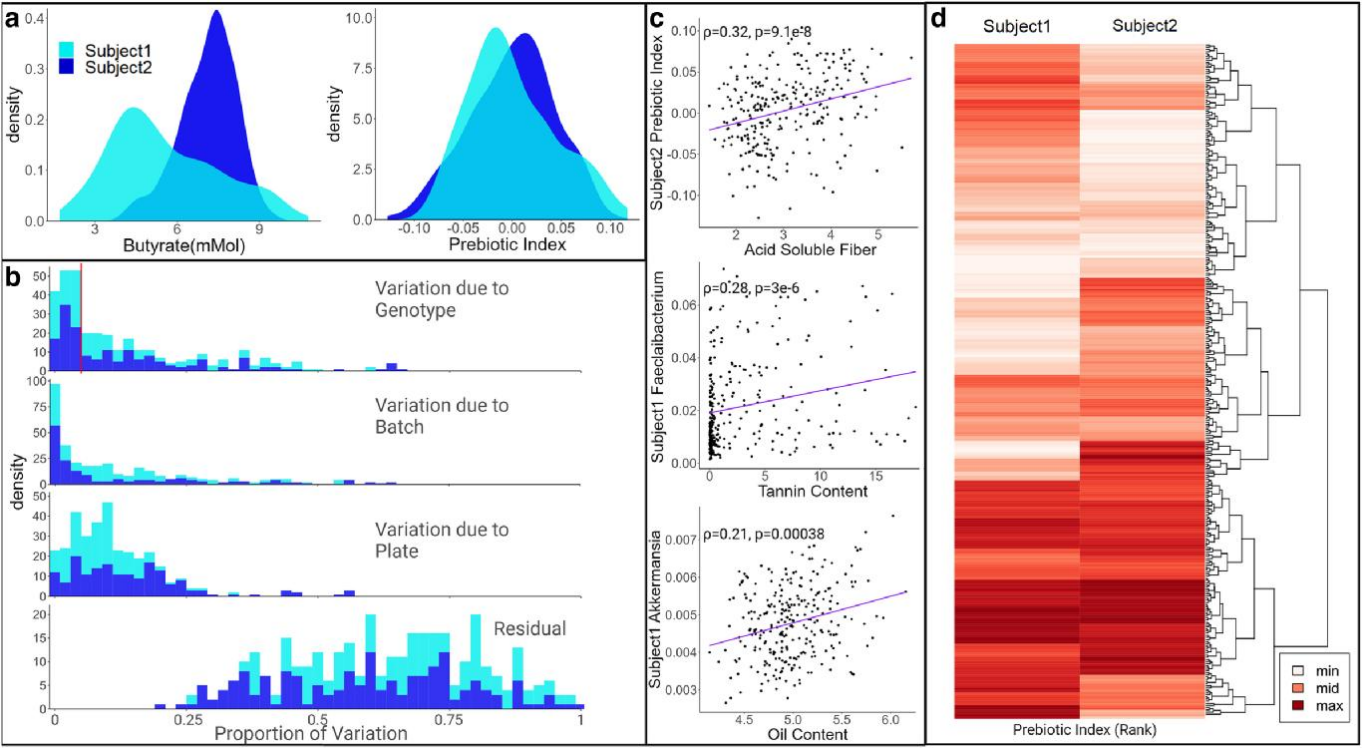
Sorghum genetics impacts microbial utilization of sorghum seed. a) Distributions of BLUEs calculated for butyrate concentrations and prebiotic indices for 340 sorghum genotypes within microbiomes from 2 human subjects. b) Variance partitioning of 342 microbiome metrics indicates that for many metrics, variation in the microbiome is explained by sorghum genotype, experimental factors, and residual error. c) Spearman correlation analyses of microbiome metrics and sorghum seed composition traits are in line with hypotheses based on existing literature, including soluble fiber–fiber-degrading organisms of the prebiotic index, tannin content, and *Faecalibacterium*, and seed oil content and lipid degradation by *Akkermansia*. Spearman's Rho (strength of association) and nonadjusted *P*-value are shown on the biplots. d) Heat map of the ranked sum abundances of prebiotic index (*x*-axis) describing each sorghum variety (*y*-axis) in the microbiomes of 2 human subjects. Higher rank (darker color indicates a higher prebiotic index). The sorghum varieties were clustered hierarchically using the hclust function in R.

Automated in vitro microbiome screening (AiMs) based phenotyping of the entire SAP resulted in a total of 2,211 individual in vitro fermentations (1,106 from the subject 1 microbiome and 1,105 from the subject 2 microbiome, excluding one sample lost during processing). Changes in the abundance of microbial taxa post-fermentation were assayed via 16S sequencing, and metabolic outcomes of fermentation were assayed using gas chromatography. A 3-step outlier identification process resulted in the removal of 28 individual AiMs reactions and 563 individual trait value observations for subject 1 and 26 samples and 650 individual trait values for subject 2. The microbiome of subject 1 comprised 3,130 individual ASVs and subject 2 comprised 2,368 ASVs. ASVs not present (at least 5 reads) in 75% of the samples from a single subject were not considered traits in downstream analyses. An estimate of heritability for each taxon was calculated and used to remove traits where variation could not be explained by variation in the genotype (*h*^2^ < 0.05, Supplementary Table 5). After filtering by abundance and heritability, the microbiome of subject 1 comprised 71 ASVs collapsed into 48 genera and 21 families, and the microbiome of subject 2 comprised 76 ASVs, collapsed into 50 genera and 19 families. Five different metrics of alpha diversity (Observed ASVs, Shannon, Simpson, Inverse Simpson, and Fisher) and 22 additional metrics that reduce the dimensionality to describe each microbiome (referred to as polymicrobial traits) were also included (10 PCs, 10 latent variables from the autoencoder analysis, and 2 versions of the prebiotic index outlined in materials and methods). We also used metabolic end products of microbial fermentation (SCFA and branched-chain fatty acids) measured post-fermentation from both subjects as quantitative traits. A prebiotic index calculated from 16S abundance data of each AiMS reaction from 2 human subjects exhibited similar distributions (Fig. 1a). In contrast, butyrate production, a microbial fermentation product assayed from the same AiMS reactions showed quite different distributions between the microbiomes, illustrating the distinctive responsiveness of the 2 diverse microbiomes to the genetic variation across the SAP (Fig. 1a).

A total of 127 traits describing the microbiome of subject 1 and 129 traits describing the microbiome of subject 2 where at least 5% of total variance was explained by differences between sorghum genotypes in a variance partitioning analysis (Fig. 1b and Supplementary Table 2) were selected for quantitative genetic analysis. The abundance of many microbes in fermented samples was correlated with variation in the sorghum seed traits, including traits that would be expected to impact specific microbial taxa and groups of microbial taxa. For example, observed expected relationships between fiber-fermenting organisms comprising the prebiotic index of subject 2 and seed fiber content (Fig. 1c, top). We also detected significant relationships between *Faecalibacterium* from subject 1 and condensed tannins (Fig. 1c, middle) that were observed in our previous genetic studies in sorghum (Yang *et al.* 2022). In addition, there is a correlation between *Akkermansia* from subject 2 and oil content (Fig. 1c, bottom) that aligns with the known utilization of unsaturated fatty acids by *Akkermansia* (Chaplin *et al.* 2015).

In many cases, the same sorghum lines produced similar functional impacts—as summarized via a prebiotic index—on polymicrobial metrics of the subject 1 and subject 2 microbiomes (Fig. 1d), despite the large baseline differences present between the 2 microbiomes (Supplementary Fig. 2). Best linear unbiased estimators (BLUEs) were calculated for each microbiome metric and are available in Supplementary data 1.

### GWAS identifies loci in sorghum genome associated with multiple changes in the composition and function of human gut microbiomes

A substantial number of trait-associated genetic markers linked to variation in the abundance of individual microbial taxa, polymicrobial traits, microbial population diversity metrics, and metabolic features (SCFAs) in each subject were identified via FarmCPU GWAS using resampling and a threshold of RMIP > 10 (Table 1). In subject 1, a total of 224 markers were associated with one or more traits, and in subject 2, a total of 329 markers were significantly associated with one or more traits (Table 1).

**Table 1.** Number of significant marker associations in the sorghum genome identified by FarmCPU (RMIP resampling value > 10) for different categories of human microbiome characteristics.

Microbiome metric	Subject 1	Subject 2
Single taxa	145	223
Diversity metrics	20	15
PolyMicrobial traits	25	83
Short-chain fatty acids	34	8

**Fig. 2.**
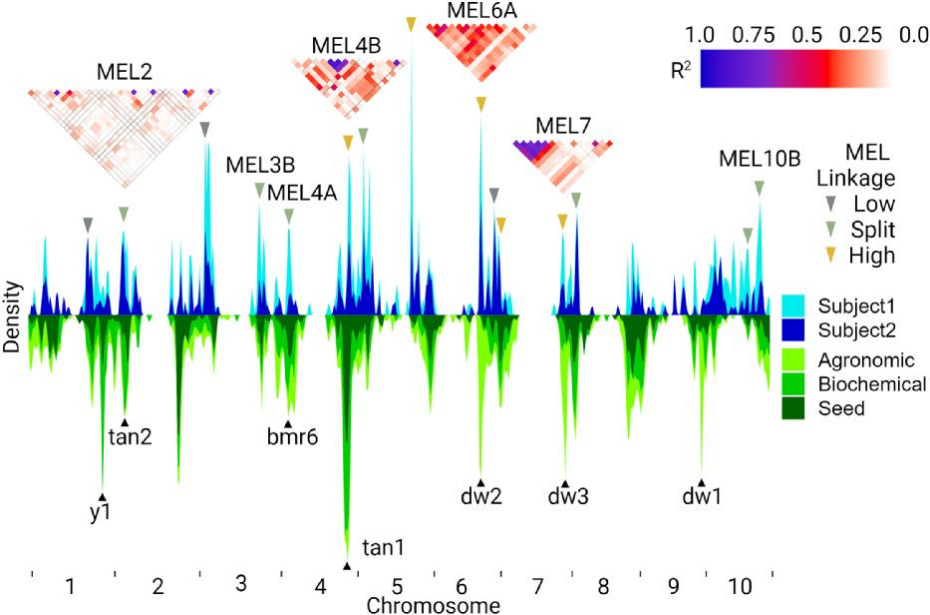
Kernel density plot of GWAS results and associated plots of LD. The *x*-axis depicts the 10 sorghum chromosomes with tick marks denoting the beginning and end of each chromosome. The *y*-axis (Density) indicates the frequency of traits associated with a physical position in the sorghum genome and is scaled based on the total number of associations in either direction for microbiome traits (blue colors at the top half of the plot) and agronomic/biochemical/seed traits (green colors at the bottom half of the plot). Linkage between significant markers is shown for 4 MEL, and degree of linkage is denoted by colored triangle heat maps. The locations of large-effect genes that have been characterized in sorghum are indicated by black triangles on the lower portion of the figure relative to their position in the genome.

Given the large number of associations, we binned the chromosomes into 75 bins each and focused on bins containing closely linked genetic markers significantly associated with at least ten microbiome metrics with at least one metric from both subjects (see Materials and methods). These are defined as MEL where genetic variation at closely linked markers in the sorghum genome has a significant influence on multiple members of the microbiome. We have previously shown that allelic effects at MEL, which were identified by the strong effects on 1 or 2 microbiomes used for AiMS phenotyping, typically are recapitulated when tested across microbiomes from multiple human subjects (Yang *et al*. 2022). MEL was identified on 9 of the 10 *S. bicolor* chromosomes and are numbered based on which chromosome they are found (Table 2). In cases where there are multiple MEL per chromosome, they are assigned a letter A–C based on marker position in ascending order. Linkage analysis among trait-associated genetic markers was used to distinguish MEL likely to correspond to a single major causal variant impacting multiple phenotypes vs 2 or more independent variants located within the same genomic interval. This is important as it can help prioritize MEL for further validation. Each MEL was defined as being in a region of low, split, or high linkage based on LD, with a split defined as two areas of high LD separated by an area of low LD (see Materials and methods). Three, 7, and 5 MEL were classified as exhibiting low, split, or high linkage, respectively (Fig. 2). Values for LD in each MEL region can be found in Supplementary Table 6 and Supplementary Fig. 3.

**Table 2.** Description of MEL including physical positions two ranges are given to MEL-assigned split linkage.

MEL	Location	Location	Linkage type	Known gene	Genes in region (expressed genes)
	(chromosome)	(Mb)			
MEL1	chr 1	54.39–55.68	Low		53 (36)
MEL2	chr 2	6.69–6.79	Split	*tan2*	7 (3)
		9.65–9.67			10* (7)
MEL3A	chr 3	7.45–8.98	Low		162 (76)
MEL3B	chr 3	52.86–52.87	Split	R?	5* (3)
		53.55–55.50			152 (79)
MEL4A	chr 4	5.88–5.89	Split	*bmr6*	14* (6)
		7.19–7.24			7* (6)
MEL4B	chr 4	61.88–63.0	High	*tan1*	142 (76)
MEL5A	chr 5	7.21–7.22	Split		3* (2)
		12.13–12.36			10 (4)
MEL5B	chr 5	5.141–5.142	High		1* (1)
MEL6A	chr 6	42.66–43.97	High	*dw2*	80 (42)
MEL6B	chr 6	51.62–57.31	Low		745 (443)
MEL6C	chr 6	59.33–60.15	High		122 (82)
MEL7	chr 7	56.56–56.99	High	*dw3*	38 (21)
MEL8	chr 8	4.31–4.46	Split		16 (5)
		5.73–5.75			3* (2)
MEL10A	chr 10	11.06–14.61	Split		138 (60)
		15.20–15.20			3* (1)
MEL10B	chr 10	48.73–48.80	Split	*dgat1*	6* (2)
		51.34–51.34			4* (2)

Known genes that may be related to seed composition in that region, the total number of genes in each region, and how many are expressed in seed. An * denotes cases where no genes were found within the initial MEL region defined by LD and an expanded window (50 additional kilobases on either side of the region) was used to identify potential candidates.

**Fig. 3.**
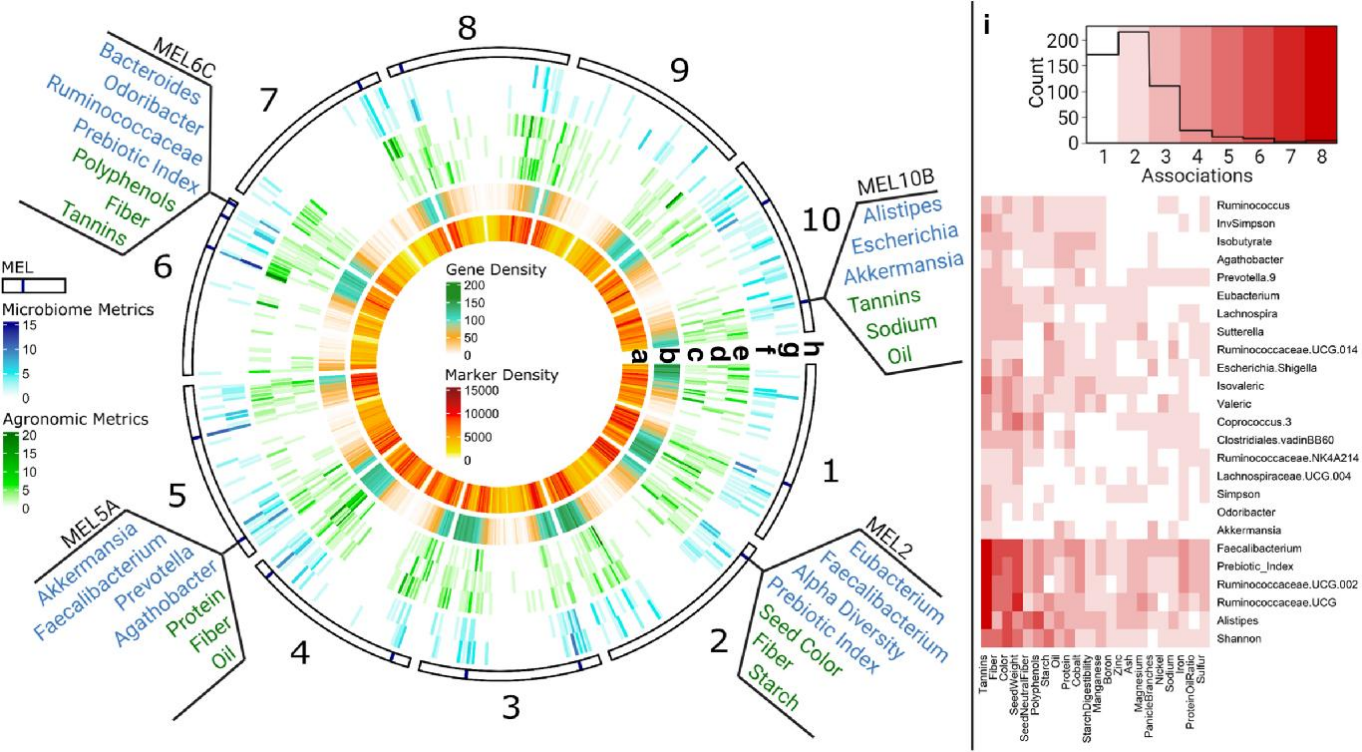
MEL locations and overlap with agronomic traits within gene-dense regions of the sorghum chromosomes. The outermost ring of the circular plot illustrates each chromosome with the positions of the MEL marked at the respective position on each chromosome. Subsets of relevant genera of the microbiome and agronomic/biochemical/seed traits mapping to MEL2, MEL5A, MEL6C, and MEL 10B are depicted at the positions on their corresponding chromosomes. Heat maps of the inner rings were colored for each of the 75 bins of ∼1 Mb created for each chromosome. Individual heat maps (from innermost to outermost) depict the marker density (a), gene density (b), the number of significant markers associated with agronomic (c), biochemical (d), and seed (e) traits, the number of significant markers associated with microbiome metrics from S1 (f) and S2 (g) and the locations of MEL (h). The heat map on the right panel (i) shows the frequency of shared associations between microbial and agronomic metrics within MEL—only metrics present in 2 or more MEL are shown.

A large number of phenotypes have been scored from the SAP (Mural *et al.* 2021). An analysis using a combination of published trait data and new human gut microbiome phenotypes collected as part of this study identified a total of 555, 350, and 426 genetic markers in the sorghum genome associated with one or more agronomic, biochemical, and seed composition traits respectively when a GWAS was conducted using the same methodology (FarmCPU < 5.8*e*–8 and RMIP > 10) and marker set employed for human microbiome traits. In several cases the genetic markers associated with agronomic/biochemical/seed traits of the SAP clustered around the locations of previously cloned and characterized large-effect sorghum genes (Fig. 2; Table 2; Supplementary Table 7). MEL was localized to marker and gene-dense regions far from centromeres (Fig. 3). In 9 MEL (2, 3B, 4A, 4B, 5A, 6A, 7, 8, and 10B), trait-associated markers for phenotypes measured from sorghum plants colocalized within MEL identified for human microbiome traits (Fig. 3; Supplementary Table 7). For example, MEL2 and MEL4B contain the *tan1* and *tan2* genes which are known to regulate the accumulation of condensed tannins in sorghum (Wu *et al.* 2012, 2019). In addition to direct association with microbial taxa phenotypes, genetic markers within these two MEL were also significantly associated with grain color, phenol abundance, and tannin concentration phenotypes (Supplementary Table 7). Notably, AiMS-based fermentation phenotyping used in previous QTL analyses of a sorghum RIL population developed from a biparental cross showed strong associations between the abundance of *Faecalibacterium* and allelic variation in *tan1* and *tan2* (Yang *et al.* 2022) and in the current GWAS of the sorghum SAP, we also found *Faecalibacterium* among the bacterial taxa from both microbiomes that were significantly associated with the genetic markers of MEL2 (*tan1*) and MEL4B (*tan2*).

Additionally, MEL10B encompasses the gene, *dgat1*, a diacylglyceroal O-acyltransferase critical in lipid biosynthesis (Zheng *et al.* 2008; Boyles *et al.* 2017). Measurements of seed oil mapped to this locus, as did the genus *Akkermansia* from subject 1. *Akkermansia muciniphila* is a known beneficial microbe whose growth has been shown to be stimulated by unsaturated fatty acids (Chaplin *et al.* 2015), implying that variation in seed oil composition may be driving variation in the *Akkermansia* abundances in the fermentations.

We also identified MEL that were not near known major effect loci but the colocalization of loci associated with microbiome and agronomic, biochemical, or seed traits (Figs. 2 and 3) at these locations provides insights to candidate mechanisms through which sorghum genetics could be affecting the human gut microbiome. For example, in MEL6C, associations with biochemical traits relating to seed phenolics and fiber content were colocalized near significant associations with microbes in the family *Ruminococcaceae* from both subjects as well as metrics describing the microbial community structure (alpha diversity and polymicrobial traits). Colocalization of these microbial traits and seed traits suggest that variation at MEL6C could affect the gut microbiome through variation in the content of polyphenols, (anthocyanins and condensed tannins), fiber content (e.g. hemicellulose content of cell walls), or an interaction between these 2 classes of seed components.

### MEL comparison with previously identified QTL

**Fig. 4.**
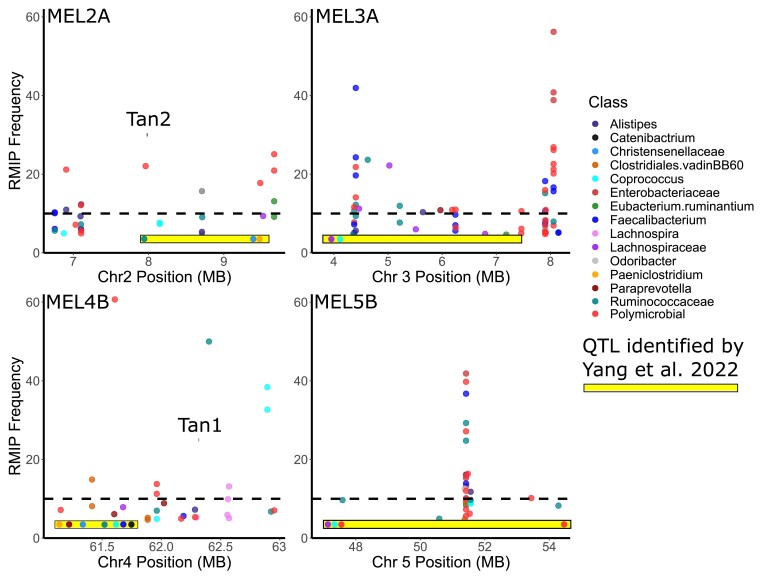
Overlap of 4 MEL and previously identified QTL and the current GWAS. Each of the plots shows the FarmCPU RMIP value for microbiome metrics (*y*-axis) zoomed into chromosome regions for each of the 4 MEL. Microbial traits showing significant associations in this region are plotted and colored by taxon. Only metrics with an RMIP value of 5 or greater are shown. ASV collapsed to genus level and any taxa that occurred only once were collapsed to family level. The QTL intervals identified from Yang *et al*. are shown as yellow boxes and microbes that were associated with these QTLs are shown as points within each box at their approximate QTL peaks.

Six QTL in sorghum describing microbiome traits were identified in our previous study where we first introduced the Automated in vitro Microbiome screening (AiMS) platform using a population of RILs from a biparental cross that were grown in a greenhouse (Yang *et al.* 2022). Of the six QTL identified, significant GWAS hits in the present study were found within or very near 4 of the 6 QTL intervals, including the *tan1* and *tan2* loci (Fig. 4). Illustrating the robustness of the AiMS-based phenotyping and the overall strategy of genetic analysis, it is important to note that we identified these 4 MEL in the 2 independent studies despite major differences in the sorghum populations being studied (SAP vs RILS from the biparental cross), despite the differences in growth conditions (field in the current study, greenhouse in the previous study) and despite the use of microbiomes from different human subjects in each study.

### Microbiome traits and agronomic, biochemical, and seed traits colocalize in MEL

To further explore the relationship between the microbial response and seed traits, we examined the frequency at which traits were observed in the same MEL. Different agronomic measurements of the same trait were combined and microbes from both subjects were collapsed at the genus level. MEL regions contained a high percentage of total significant markers for the plant traits, across all associated markers, 21.7, 22.5, and 15.9% of biochemical, seed, and agronomic traits, respectively, were contained within the boundaries of the identified MEL. Of microbial traits associated with multiple MEL, several co-occur with sorghum phenotypes describing the seed traits, including tannins, fiber content, seed color, and seed weight (Fig. 3i) in up to 8 of the identified MEL. At least one tannin measurement was associated with a marker near 11 of the 15 identified MEL, and the co-occurrence of tannin with tannin-related organisms in the *Ruminococcaceae* family and alpha diversity was expected. In the case of seed fiber, color, and weight, these traits were present in 7, 8, and 9 total MEL respectively, and in almost fall they co-occur with specific genera: fiber and *Faecalibacterium*, color and *Alistipes*, weight and *Ruminococcaceae.UCG* and *Coprococcus*. Co-occurrence of these agronomic traits and microbiome traits in the same MEL implies that breeding and selection for traits such as seed color and seed size could indeed have significant impacts on the human gut microbiome.

### Investigation of MEL6A

Among the MEL where we observed colocalization of microbiome and agronomic traits, a promising MEL was selected for a molecular complementation study from MEL grouped into the “high linkage” category because it is most probable in these MEL a single marker which may translate to 1 or a small number of nearby gene(s). MEL6A was particularly intriguing as several agronomic and seed traits (seed weight, harvest index, and panicle area) map to the same locus along with a number of individual microbiome traits (*Faecalibacterium* from both subjects, *Escherichia* from subject 1, and *Coprococcus* plus other members of the *Lachnospiraceae* from subject 2) and polymicrobial traits (PC2 in both subjects, LV2 in subject 1 and prebiotic index in subject 2).

**Fig. 5.**
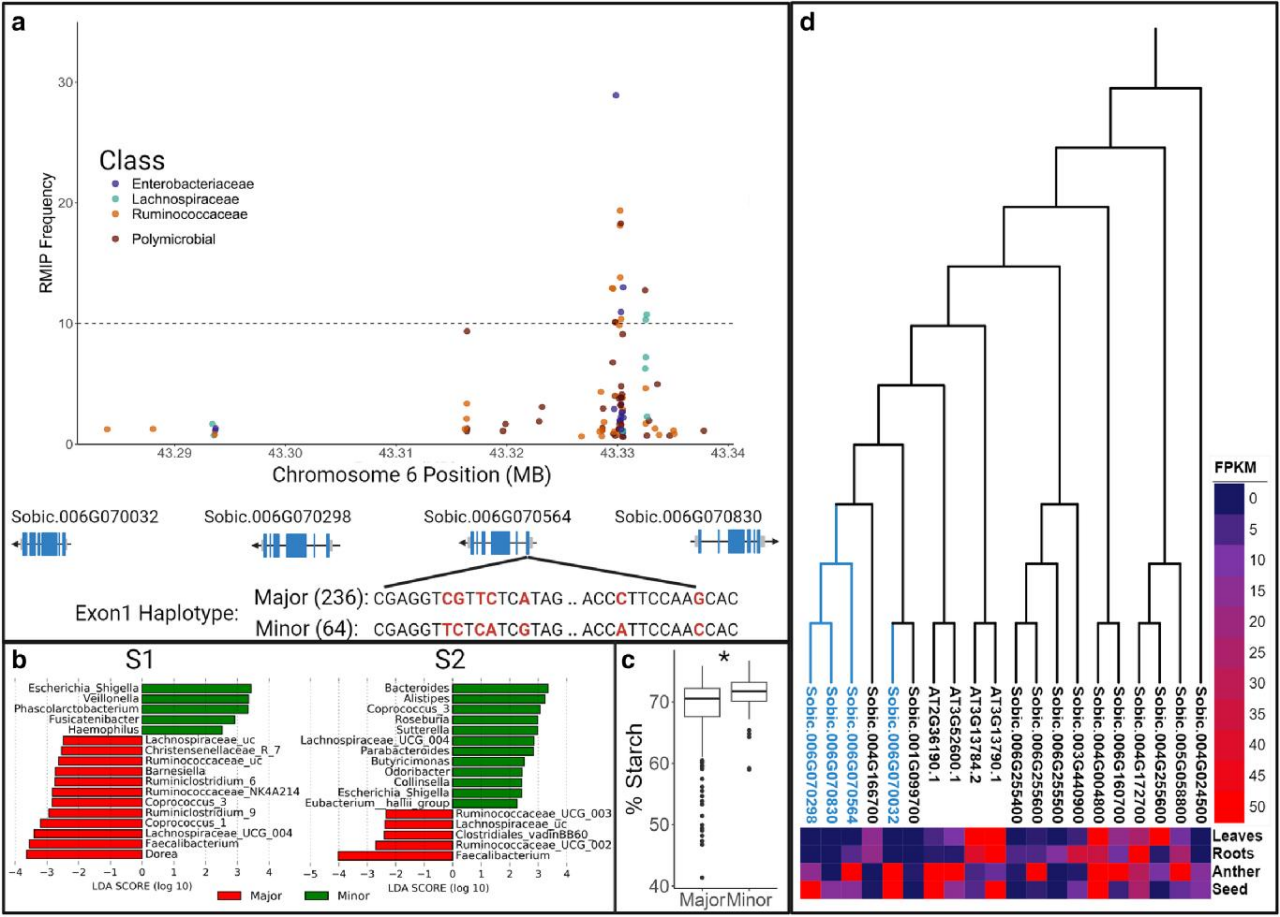
Dissection of MEL6A. The RMIP values for taxa significantly associated with MEL6A colored by family or polymicrobial within the 43.2–43.34 Mb region of chromosome 6 (a). Markers most frequently associated with microbiome metrics within MEL6A were within a tandem array of sorghum genes encoding cell wall invertases on the same *x*-axis. Haplotypes in perfect LD with the major and minor allele at the GWAS peak are shown below the Sobic006G070564 CWIN gene with numbers of lines in the SAP with major and minor alleles in parentheses (a). Results of LEfSE analysis taxa in S1 and S2 show significant differences between lines carrying the major vs minor allele at the GWAS peak (b). Box and whisker plot shows the % starch content of seeds from lines carrying the major or minor alleles at GWAS peak (c). A dendrogram developed from protein sequence similarity paired with a heat map of gene expression in the form of Fragments per kilobase of transcript (FKPM) illustrates genes that are highly expressed in sorghum seed (blue leaves on the dendrogram) are more similar in sequence to a well-studied set of *Arabidopsis* cell wall invertases (neighboring clade) than other copies found in sorghum (d).

Centered within MEL6A on the sorghum genome is a tandem array of four cell wall invertase (CWIN) genes located ∼43 Mb from the start of sorghum chromosome 6 (Fig. 5a). Two of the significantly trait-associated genetic markers were S06_43329706 and S06_43330339. These markers were immediately adjacent to the tandem array of CWIN genes and they are associated with the most microbial metrics within this MEL, including subject 2 prebiotic index and multiple groups within the families, *Lachnospiraceae*, *Ruminococcaceae*, and *Enterobacteriacae* from both human subjects (Fig. 5a). Sorghum has a total of 16 genes annotated as encoding a b-fructofuranosidase, otherwise known as invertase. However, only 8 of these genes, including the 4 CWIN within MEL6A, are expressed in developing sorghum grain. Moreover, the MEL6A CWIN appears to be exclusively expressed in developing seeds whereas the 4 other seed-expressed invertases are also expressed in leaf and/or root tissue (Fig. 5d). The MEL6A CWIN are also part of a 6 gene subfamily in sorghum that appear to be orthologous to a 4 gene subfamily of CWIN in *Arabidopsis* which also exhibits substantial seed-specific expression, the other 10 sorghum invertases share more peptide similarity with vacuolar invertases and are likely not cell wall-bound. Cell wall invertases in other plant species are known to play important roles in sucrose metabolism, and loss of function mutations of these genes can be pleotropic-producing phenotypes influencing the formation of fructooligosaccharides, flower and seed development, seed size, starch accumulation, starch composition, stress response, and the phenylpropanoid pathway (Baumert *et al.* 2001; Chourey *et al.* 2012; Nishanth *et al.* 2018). While the peak genetic markers identified by the FarmCPU resampling-based GWAS were located in intergenic space between 2 of the tandem gene copies, a number of nonsynonymous polymorphisms within coding sequences were in high LD with the peak genetic markers. Four nonsynonymous polymorphisms in perfect linkage—consisting of a three base pair deletion, a 3 bp insertion, and 2 point mutations—within the first exon of Sobic.006G070564 were in tight linkage with the peak markers identified in the GWAS analysis, forming 2 distinct haplotypes at this locus. LefSE analysis of microbial taxa at the genus level, using haplotype at this position as the explanatory variable, identified significant changes (*P*-value <0.05) in the abundance of several genera from both subjects. Similar to the GWAS data, the abundance of *Escherichia* was higher in samples from both subjects fermented with the grain of sorghum varieties carrying the minor haplotype, and in both subjects, the abundance of *Faecalibacterium* and other taxa (members of the *Ruminococcaceae* and *Lachnospiraceae*) was greater in samples fermented with the grain of sorghum varieties carrying major haplotype (Fig. 5b). Total starch content was also significantly higher (*P*-value = 0.0025, effect = 2.04%) among sorghum varieties carrying the minor haplotype at this locus (Fig. 5c), which is somewhat unexpected since the amylolytic bacterial taxa mapping here are more abundant in fermentations from lines carrying the major allele. However, CWIN is pleiotropic, affecting not only total starch but also starch composition (amylopectin:amylose ratios), and constitutive expression of CWIN in maize can increase total starch while also reducing amylose content (Li *et al.* 2013). This matches well with our microbial phenotypes as *Faecalibacterium* and other amylolytic species of the *Ruminococcaceae* and *Lachnospiraceae* which preferentially ferment amylose and map to MEL6A are elevated in lines carrying the major allele. Consequently, 1 would hypothesize that the minor allele haplotype in Sobic006G070564 increases starch content in the seed but reduces its amylose content, having a major effect on the gut microbiome.

### Variation at the MEL6A locus produces consistent effects across diverse human microbiomes

**Fig. 6.**
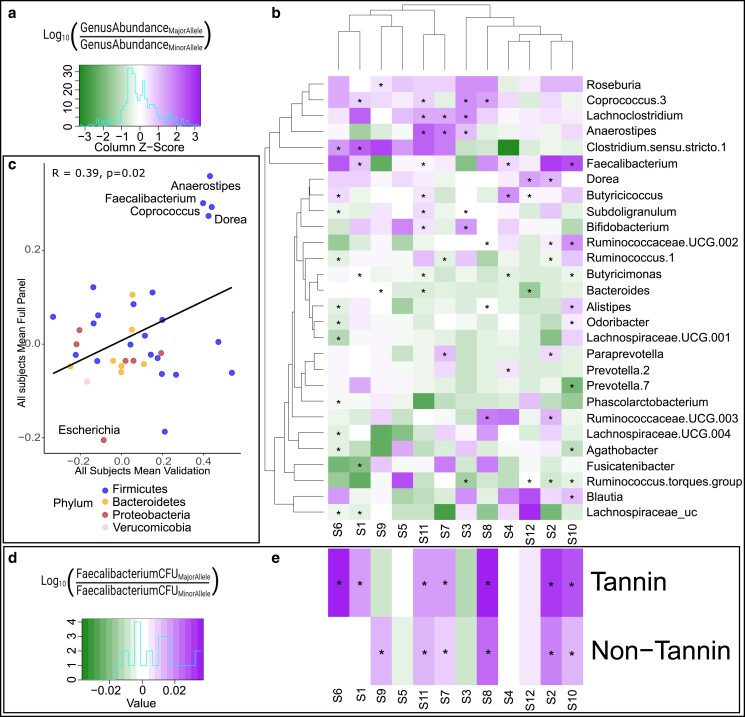
Validation of allelic effects at MEL6A. The equation and heat map legend (a) for log ratios of top genera from the microbiomes of 12 human subjects treated with the major haplotype by minor haplotype at the MEL6A allele within the tannin-containing pools. The heat map is colored based on *Z*-score (log-fold change value normalized by row) and statistically significant *P*-values based on Pearson correlation are indicated for the relevant taxon/subject with an asterisk. The heat map was clustered by hierarchical 2D clustering with the hclust function in *R* (b). Scatter plot of log2-fold change in genera in AiMS reactions of major haplotype/minor haplotype for all lines from the SAP mapping with S1 and S2 microbiomes (*x*-axis) and pools of lines carrying major vs minor MEL6A allele fermented by 12 different human subjects (*y*-axis). The *x* and *y* values for each genus are the average fold change of the genus in the S1 and S2 microbiome for each SAP line (*x*-axis) and the average fold change of the same genus in all 12 subjects for the major and minor MEL6a pools (*Y*-axis). Each genus is colored according to its corresponding phylum the Pearson correlation coefficient and *P*-values are shown (c). The equation and heat map legend (d) for the mean values of Log ratios of *Faecalibacterium* from the microbiomes of 12 human subjects quantified by qPCR treated with the major haplotype by minor haplotype at the MEL6A allele in both tannin and non-tannin pools (e). Asterisks denote significant *P*-values from Wilcoxon tests comparing the abundance of *Faecalibacterium* in sorghum lines that contain the major allele vs lines that contain the minor allele, *P*-value <0.05.

The effect of tannins (MEL2 and MEL4B) and the haplotype group at MEL6A, drove strong and similar responses in microbiome abundance profiles, including organisms that are members of the family Lachnospireace family and *Faecalibacterium* genus and we have previously characterized those responses (Yang *et al.* 2022). Microbiome abundance profiles similar to those observed at the *tan1* and *tan2* loci were observed at MEL6A though there are no known genes that are part of the proanthocyanidin (tannin biosynthesis) pathway within the MEL6A region, and indeed, it seems that the phenotype could be driven by starch composition. To determine if the microbiome effects of tannin content (MEL2 and MEL4B) and MEL6A are additive or due to a possible interaction relationship between loci, sorghum lines were pooled first by an allele at MEL6A peak and then by presence/absence of tannins. Each pool was processed in the AiMs platform and screened across the microbiomes of 12 human donors, including microbiomes from subject 1 and subject 2 (see Materials and methods). Both the MEL6A allele at GWAS peak and tannin content impacted microbial community structure in several subjects as indicated in an analysis of beta diversity, where significant differences by allele in 10 of the 12 subjects were observed (Supplementary Fig. 4). Across the top 30 genera present in all 12 human subjects based on 16S sequencing, several genera showed significant differences based on MEL6A allele of the pooled lines (*P*-value <0.05). Although the effect of MEL6A allele was microbiome-dependent (e.g. different organisms were observed responding to the MEL6A genotype effect in all subjects), allelic effects of MEL6A nonetheless affected multiple taxa in each microbiome (Fig. 6a and b). In an analysis of the average log2-fold change in all subjects, the response of many taxa was as expected from the initial analysis, with higher abundances of *Anerostipes*, *Faecalibacterium*, *Coprococcus*, and *Dorea* in lines and pools that contain the major haplotype, and lower abundances of *Escherichia* (Fig. 6c). The response of many genera was consistent across experiments even when compiling subjects in the log2-fold change analysis, (Pearson correlation, *R* = 0.39, *P*-value = 0.02). In 3 of 12 subjects’ microbiomes, the abundance of *Faecalibacterium* was significantly higher in the MEL6A major allele haplotype pools (Fig. 6a and b). To further investigate the effect of the MEL6A haplotype on *Faecalibacterium*, the absolute abundance of *Faecalibacterium* was quantified using qPCR for microbiomes from each of the 12 subjects treated with the haplotype pools. With this more quantitative measure, the amount of *Faecalibacterium* was significantly higher in microbiomes treated with the major allele in 8 of the 12 subjects (Fig. 6d and e). In an analysis of variance (ANOVA) of absolute abundance of *Faecalibacterium* quantified by qPCR, with factors subject, presence/absence of tannins, allele at MEL6A, and all interactions between them, all factors were highly significant (*P*-value <0.0005) except the interaction term for tannin and MEL6A allele (Supplementary Table 8). Thus, while the allele at MEL6A and the effects of allelic variation at MEL2 and MEL4B on tannin content all affect the growth of *Faecalibacterium*, they appear to do so independently and thus likely affect this genus through variation in different components of the seed (e.g. tannins and starch composition).

## Discussion

### Repeatability of AiMS-based mapping data across multiple populations

In this study, we identified a total of 15 MEL distributed across the sorghum genome where sorghum genetic variation produces substantial impacts on diverse human gut microbiomes. This included reidentification and more precise localization of 4 broad QTL described in a previous study conducted using a biparental sorghum mapping population (Yang *et al.* 2022). It is highly probable these overlapping loci correspond to variation in the same gene(s) in the loci, however, it is also possible that they are driven by variation in different but closely linked genes. The increased number of MEL identified in this study is consistent with the better presentation of genetic and phenotypic diversity provided by association panels relative to recombinant inbred populations, and the more precise localization is consistent with the larger number of recombination events captured by association mapping relative to structured populations such as recombinant inbred populations. At the same time, the reidentification of the MEL described in a study conducted using a different sorghum population grown in a different environment—Yang *et al.* (2022) used grain from individual plants grown in greenhouses, our study uses grain from small plot trials grown in the field—suggests that the MEL we identify are likely to generalize across sorghum populations and provide consistent effects on human gut microbiomes when sorghum is grown in different parts of the globe and different years, increasing the feasibility of breeding sorghum for beneficial microbial outcomes. Of the 15 MEL from the current study, 4 (MEL2, MEL3A, MEL4B, and MEL5B) correspond to the same general location as 4 of the 6 total MEL detected in the Yang *et al*. study (Fig. 4). Thus, genetic diversity within the SAP not only validated previous findings, it also further extended the catalogue of loci in *S. bicolor* where genetic variation has multiple effects on the microbiome. In both studies, tannin content and microbes belonging to the families Ruminococcaceae and Lachnospiraceae mapped with overlapping peaks to MEL2 and MEL4B, which correspond to the *tan2* and *tan1* regulatory genes, respectively, which control the synthesis of condensed tannins. Remarkably, though we used microbiomes from different human subjects in the 2 studies, the microbiome phenotypes of MEL2 and MEL4B across multiple human subjects consistently showed major effects of variation on *Faecalibacterium* and other members of the Ruminococcaceae along with members of the Lachnospiraceae. Thus, even in very distinct genetic backgrounds (SAP vs BTx623 X IS3620C RILs), variation at *tan1* and *tan2* imparts conserved signatures on the human gut microbiome across diverse human subjects. Other genes in sorghum where variation has been shown to have a large phenotypic effect were located near MEL including *bmr6*, *dw2*, and *dw3* (Fig. 2). The brown midrib mutant 6 (*bmr6*) gene is localized near MEL4A and is involved in the biosynthesis of lignin. There is a distinct possibility this gene is related to the microbial response associated with MEL4A, but the relationship has yet to be explored. The dwarf genes *dw2* and *dw3* that align with MEL6A and MEL7, respectively, have been connected to plant height and other physiological shoot phenotypes in sorghum. While it is unlikely that the dwarf genes are directly related to the microbiome phenotype observed, selection for variants in these genes likely expanded the frequency of mutations in nearby genes which are causal for the microbial response.

### The human gut microbiome as a diverse set of phenotypes to discover novel seed traits

In addition to validating previous findings, it is worth noting that our emphasis on MEL, which affects multiple microbial taxa from different microbiomes, focuses the genetic analysis on loci where variation can have a major effect across human populations. As observed in our previous study, when allelic effects of the MEL are tested across a larger number of human microbiomes they generally show significant responses, though taxonomic signatures of the responses are not identical, there are often similar responses of shared taxa across multiple microbiomes (Yang *et al.* 2022). Such reproducibility was illustrated in the current study from the similar allelic effects of MEL6A on the absolute abundance of *Faecalibacterium* (Fig. 6d and e) and members of the Lachnospiraceae across the S1 and S2 microbiomes and many of the 10 additional human subjects that were tested with seed pools from lines carrying the major vs minor MEL6A allele (Fig. 6a). How well the in vitro reactivity predicts the effects that would be observed in human feeding studies remains uncertain as no study has directly compared microbiome outcomes of AiMS batch fermentations to feeding studies in humans. However, a single study measuring microbiome outcomes of batch fermentations and feeding studies in humanized mice (using the same microbiomes as the batch fermentations) showed significant treatment effects of feeding wildtype vs waxy sorghum could be detected in vitro (Yang *et al.* 2023). While the in vitro and in vivo microbiome signatures showed only limited overlap, the treatment effect of reduced resistant starch in waxy vs wildtype is of primary interest. Similarly, the signature effects of condensed tannins in sorghum on *Faecalibacterium* and other members of the Ruminococcaceae that we have detected in vitro in this study and our previous study is also observed in feeding studies of swine and poultry (Choy *et al.* 2014; Díaz Carrasco *et al.* 2018; Brugaletta *et al.* 2020). Thus, we believe strong signatures of genetic variation in sorghum on the microbiome detected in vitro (e.g. MEL) are likely to translate to significant microbiome outcomes in vivo, although the taxonomic signatures of the outcomes may vary. Consequently, despite the individuality of the gut microbiome in humans and despite the use of in vitro conditions for mapping, emphasis on MEL enables us to use relatively small numbers of microbiomes for mapping across large numbers of sorghum genotypes, ultimately allowing us to survey a significant proportion of genetic variation present within the crop and identify candidate loci that are likely to broadly affect human gut microbiomes.

The 11 new MEL identified in this study also had significant associations with multiple organisms and/or polymicrobial traits from the microbiomes of both subjects used in the phenotyping and we fully expect genetic variation at these loci to also have major effects across microbiomes of additional human subjects. While we observed many overlapping peaks for microbial groups at different levels of taxonomic resolution with polymicrobial traits, there were some instances in which polymicrobial traits mapped at loci where no single taxa mapped (Supplementary Fig. 5). Such loci may correspond to variation that is associated with complex patterns of taxonomically unrelated organisms such as functional groups of microbes that act together ecologically in response to substrate variation. In addition, we also found significant associations with the Transformed Prebiotic Indices (PreInd T and PreInd BT) with nine of the 15 MEL (Supplementary Table 4). The PreInd BT, which includes only beneficial organisms, was captured in 8 MEL, suggesting that this may be a better metric for genetic analyses. While the PreInd BT is a relatively simple index we developed as a proof-of-concept, we note that all eight MEL with significant associations with the PreInd BT had significant colocalizing associations with 2 or more beneficial taxa that are included in the PreInd BT formula (Supplementary Tables 4 and 8). There may be merit in using more sophisticated methods for estimating a singular “health-promoting” index, such as the Gut Microbiome Health Index (GHMI) (Gupta *et al.* 2020) in future studies, however, our PreInd BT, which contains a subset of beneficial taxa from the GHMI list nonetheless may be a useful tool to study the genetic basis of beneficial microbial variation.

### Colocalization of microbiome traits and agronomic/biochemical traits

In addition to multiple microbiome traits mapping to each MEL, we also found that multiple agronomic, seed, or biochemical traits mapped within each of the MEL. Interestingly, there was only slight enrichment for biochemical and seed traits as roughly 21.7 and 22.5%, respectively of significantly associated markers are within MEL compared to 15.9% markers associated with agronomic traits. However, as we illustrated at MEL6A and MEL10B, the colocalized microbiome and seed traits, along with known genetic variation at the locus, facilitated hypothesis generation about causal variants and mechanisms through which such variation impacts the microbiome. We note that significant associations for traits associated with seed color (tannins, polyphenols, etc.), seed size/weight, and the content of seed starch, total fiber, and oil content colocalized along with microbiome phenotypes to nearly every MEL, apart from MEL3A (Supplementary Table 7). As is illustrated with the known pleiotropic effects of variation at *tan1* on tannins, seed color, starch content, and microbiome (Yang *et al.* 2022), genetic variation driving phenotypes corresponding to the MEL can be quite pleiotropic. A major implication from such pleiotropy is that breeding for agronomic or seed traits (e.g. seed size, color, weight, etc.) that are related to desirable agricultural characteristics may also have profound effects on the human gut microbiome. Even if the variation within a MEL that affects agronomic features and microbiome features is distinct, the high degree of linkage would still imply that breeding and selection for these agronomic features could still have pleiotropic effects on the human gut microbiome. We also observed MEL where few known agronomic, biochemical, or seed traits colocalized (Fig. 3). At these loci, the seed components affecting the microbiome are unknown and this experiment provides an opportunity to elucidate novel seed traits in sorghum.

### Candidate genes and hypothesis generation for effects of MEL on the microbiome

Colocalization of seed traits and microbiome traits at a MEL can help drive hypotheses for candidate genes/pathways that drive the seed and microbial phenotypes. Beyond the *tan1* and *tan2* loci, we prioritized MEL with high linkage since it is more likely that the microbial phenotypes would be driven by variation from a single gene. This led us to the high linkage MEL6A and the tandem array of cell wall invertases (CWIN). b-Fructofuranosidase activity (sucrose → glucose + fructose) from CWIN can influence a wide array of plant functions such as growth and development, carbon/nutrient partitioning, and responses to biotic and abiotic stress. Thus, if the CWIN haplotype associated with the minor allele in MEL6A is responsible for the microbiome and seed traits, it may be manifested through a number of mechanisms. We initially tested total fructans across seeds with the major vs minor allele and CWIN haplotype content at MEL6A and found no statistically significant differences (data not shown). We did, however, detect significant effects on total starch content and many of the microbial taxa mapping to MEL6A are known amylolytic organisms that would be expected to show differences if starch content were driving the microbial phenotype. More work is necessary to determine if it is composition of the starch (e.g. amylose:amylopectin ratios) is driving the phenotype, but expression of CWIN in maize can drive very similar starch phenotypes (elevated total starch but with decreased amylose content) that would be expected to drive the microbial phenotypes that are associated with MEL6A. Exploration of other MEL identified in this study, especially in regions of high or split linkage may lead to the identification of causative genes and mechanisms in sorghum that could be leveraged to improve its (or related species) nutritional quality.

### The human gut microbiome as an agnostic approach to identification of traits in food crops that can affect human health and nutrition

While we can currently only generate mechanistic hypotheses around a small number of the MEL, the nature of the MEL themselves (e.g. major effects on multiple microbes and the ability to detect allelic effects of MEL across different human subjects) illustrates the strength of this approach for identifying novel plant traits affecting human health and nutrition. Unlike mapping specific biochemical traits, the use of different features of the human gut microbiome as a complex combination of traits (e.g. abundances of individual taxa, PCs comprising multiple taxa, prebiotic indices) allows us to agnostically interrogate the effects of plant genetic variation on a wide variety of traits that can have a major impact on human health. Moreover, once MEL is discovered, one can reduce the phenotypic analysis to individual microbes, groups of microbes, and microbial metabolites (e.g. SCFA) to simplify phenotypic analysis. While the exact biochemical pathway that drives a MEL may not be known, the microbial trait itself (through AiMs-based fermentations) can be used in an efficient and high-throughput manner to facilitate further genetic analysis to localize causal variants and downstream breeding to improve the trait.

## Supplementary Material

jkae145_Supplementary_Data

## Data Availability

The DNA sequencing reads for this study are available in the NCBI SRA database as project accession PRJNA1012736. All ASVs were assigned with taxonomic information using the taxonomy classifier SILVA database (Quast *et al.* 2013). All data are available in the main text or the Supplementary materials. BLUEs calculated for microbiome metrics and sorghum agronomic data are included in Supplementary data 1. Complete GWAS output of all traits is included in Supplementary data 2. The qPCR output of *Faecalibacterium* in the MEL6A validation study is included in Supplementary data 3. The bioinformatic pipeline designed for this study is available at https://github.com/natekorth/SAP. Supplemental material available at G3 online.
