# Supplementary material for: Genomic co-localization of variation affecting agronomic and human gut microbiome traits in a meta-analysis of diverse sorghum
Source: G3 (Bethesda). 2024 Jul 9;14(9):jkae145. doi: 10.1093/g3journal/jkae145 (PMC11373648; doi:10.1093/g3journal/jkae145)
Supplement: jkae145_Supplementary_Data [file jkae145_supplementary_data.zip › References_for_Supplemental_Material_G3-2024-405129.docx]

**References for supplemental material:**

Aguirre, M., A. Eck, M. E. Koenen, P. H. M. Savelkoul, A. E. Budding *et al.*, 2016 Diet drives quick changes in the metabolic activity and composition of human gut microbiota in a validated in vitro gut model. Res. Microbiol. 167: 114–125.

Ai, D., H. Pan, X. Li, Y. Gao, G. Liu *et al.*, 2019 Identifying gut microbiota associated with colorectal cancer using a zero-inflated lognormal model. Front. Microbiol. 10: 826.

Astbury, S., E. Atallah, A. Vijay, G. P. Aithal, J. I. Grove *et al.*, 2020 Lower gut microbiome diversity and higher abundance of proinflammatory genus Collinsella are associated with biopsy-proven nonalcoholic steatohepatitis. Gut Microbes 11: 569–580.

Benítez-Páez, A., E. M. Gómez del Pugar, I. López-Almela, Á. Moya-Pérez, P. Codoñer-Franch *et al.*, 2020 Depletion of Blautia Species in the Microbiota of Obese Children Relates to Intestinal Inflammation and Metabolic Phenotype Worsening. mSystems 5:.

Boyles, R. E., E. A. Cooper, M. T. Myers, Z. Brenton, B. L. Rauh *et al.*, 2016 Genome-Wide Association Studies of Grain Yield Components in Diverse Sorghum Germplasm. Plant Genome 9: plantgenome2015.09.0091.

Boyles, R. E., B. K. Pfeiffer, E. A. Cooper, B. L. Rauh, K. J. Zielinski *et al.*, 2017 Genetic dissection of sorghum grain quality traits using diverse and segregating populations. Theor. Appl. Genet. 130: 697–716.

Brahe, L. K., E. Le Chatelier, E. Prifti, N. Pons, S. Kennedy *et al.*, 2015 Specific gut microbiota features and metabolic markers in postmenopausal women with obesity. Nutr. Diabetes 2015 56 5: e159–e159.

Brown, P. J., W. L. Rooney, C. Franks, and S. Kresovich, 2008 Efficient mapping of plant height quantitative trait loci in a sorghum association population with introgressed dwarfing genes. Genetics 180: 629–637.

Castaño-Rodríguez, N., A. P. Underwood, J. Merif, S. M. Riordan, W. D. Rawlinson *et al.*, 2018 Gut microbiome analysis identifies potential etiological factors in acute gastroenteritis. Infect. Immun. 86: 60–78.

Chang, S. C., M. H. Shen, C. Y. Liu, C. M. Pu, J. M. Hu *et al.*, 2020 A gut butyrate‑producing bacterium Butyricicoccus pullicaecorum regulates short‑chain fatty acid transporter and receptor to reduce the progression of 1,2‑dimethylhydrazine‑associated colorectal cancer. Oncol. Lett. 20: 1–1.

Chen, Y. J., H. Wu, S. Di Wu, N. Lu, Y. T. Wang *et al.*, 2018 Parasutterella, in association with irritable bowel syndrome and intestinal chronic inflammation. J. Gastroenterol. Hepatol. 33: 1844–1852.

Choi, S., Y. J. Hwang, M. J. Shin, and H. Yi, 2017 Difference in the gut microbiome between ovariectomy-induced obesity and diet-induced obesity. J. Microbiol. Biotechnol. 27: 2228–2236.

Companys, J., M. J. Gosalbes, L. Pla-Pagà, L. Calderón-Pérez, E. Llauradó *et al.*, 2021 Gut microbiota profile and its association with clinical variables and dietary intake in overweight/obese and lean subjects: A cross-sectional study. Nutrients 13: 2032.

Cuffaro, B., A. L. W. Assohoun, D. Boutillier, L. Súkeníková, J. Desramaut *et al.*, 2020 In Vitro Characterization of Gut Microbiota-Derived Commensal Strains: Selection of Parabacteroides distasonis Strains Alleviating TNBS-Induced Colitis in Mice. Cells 9: 2104.

Daillère, R., M. Vétizou, N. Waldschmitt, T. Yamazaki, C. Isnard *et al.*, 2016 Enterococcus hirae and Barnesiella intestinihominis Facilitate Cyclophosphamide-Induced Therapeutic Immunomodulatory Effects. Immunity 45: 931–943.

Din, A. U., A. Hassan, Y. Zhu, K. Zhang, Y. Wang *et al.*, 2020 Inhibitory effect of Bifidobacterium bifidum ATCC 29521 on colitis and its mechanism. J. Nutr. Biochem. 79: 108353.

Eeckhaut, V., J. Wang, A. Van Parys, F. Haesebrouck, M. Joossens *et al.*, 2016 The probiotic butyricicoccus pullicaecorum reduces feed conversion and protects from potentially harmful intestinal microorganisms and necrotic enteritis in broilers. Front. Microbiol. 7: 1416.

Everard, A., C. Belzer, L. Geurts, J. P. Ouwerkerk, C. Druart *et al.*, 2013 Cross-talk between Akkermansia muciniphila and intestinal epithelium controls diet-induced obesity. Proc. Natl. Acad. Sci. U. S. A. 110: 9066–9071.

Figueroa-González, I., G. Rodríguez-Serrano, L. Gómez-Ruiz, M. García-Garibay, and A. Cruz-Guerrero, 2019 Prebiotic effect of commercial saccharides on probiotic bacteria isolated from commercial products. Food Sci. Technol. 39: 747–753.

Gomez-Arango, L. F., H. L. Barrett, S. A. Wilkinson, L. K. Callaway, H. D. McIntyre *et al.*, 2018 Low dietary fiber intake increases Collinsella abundance in the gut microbiota of overweight and obese pregnant women. Gut Microbes 9: 189–201.

Gupta, A., D. B. Dhakan, A. Maji, R. Saxena, V. P. P.K. *et al.*, 2019 Association of Flavonifractor plautii, a Flavonoid-Degrading Bacterium, with the Gut Microbiome of Colorectal Cancer Patients in India. mSystems 4:.

Hanifi, G. R., H. Samadi Kafil, H. Tayebi Khosroshahi, R. Shapouri, and M. Asgharzadeh, 2021 Bifidobacteriaceae family diversity in gut microbiota of patients with renal failure. Arch. Razi Inst. 76: 521–528.

He, B., Y. Bai, L. Jiang, W. Wang, T. Li *et al.*, 2018 Effects of oat bran on nutrient digestibility, intestinal microbiota, and inflammatory responses in the hindgut of growing pigs. Int. J. Mol. Sci. 19: 2407.

Henneke, L., K. Schlicht, N. A. Andreani, T. Hollstein, T. Demetrowitsch *et al.*, 2022 A dietary carbohydrate–gut Parasutterella–human fatty acid biosynthesis metabolic axis in obesity and type 2 diabetes. Gut Microbes 14:.

Hou, D., Q. Zhao, L. Yousaf, J. Khan, Y. Xue *et al.*, 2020 Consumption of mung bean (Vigna radiata L.) attenuates obesity, ameliorates lipid metabolic disorders and modifies the gut microbiota composition in mice fed a high-fat diet. J. Funct. Foods 64: 103687.

Kim, S., R. Goel, A. Kumar, Y. Qi, G. Lobaton *et al.*, 2018 Imbalance of gut microbiome and intestinal epithelial barrier dysfunction in patients with high blood pressure. Clin. Sci. 132: 701–718.

Lam, Y. Y., C. W. Y. Ha, C. R. Campbell, A. J. Mitchell, A. Dinudom *et al.*, 2012 Increased gut permeability and microbiota change associate with mesenteric fat inflammation and metabolic dysfunction in diet-induced obese mice. PLoS One 7: e34233.

Lanthier, N., J. Rodriguez, M. Y. Nachit, S. Hiel, P. Trefois *et al.*, 2021 Lower abundance of Clostridium sensu stricto is associated with liver steatosis and fibrosis severity in a prospective cohort of obese patients with metabolic dysfunction-associated fatty liver disease. Acta Gastroenterol. Belg. 84: A20.

Lasky, J. R., H. D. Upadhyaya, P. Ramu, S. Deshpande, C. T. Hash *et al.*, 2015 Genome-environment associations in sorghum landraces predict adaptive traits. Sci. Adv. 1:.

Li, Q., Y. Chang, K. Zhang, H. Chen, S. Tao *et al.*, 2020 Implication of the gut microbiome composition of type 2 diabetic patients from northern China. Sci. Rep. 10: 1–8.

Liu, Y., L. Zhang, X. Wang, Z. Wang, J. Zhang *et al.*, 2016 Similar Fecal Microbiota Signatures in Patients With Diarrhea-Predominant Irritable Bowel Syndrome and Patients With Depression. Clin. Gastroenterol. Hepatol. 14: 1602-1611.e5.

Machiels, K., M. Joossens, J. Sabino, V. De Preter, I. Arijs *et al.*, 2014 A decrease of the butyrate-producing species roseburia hominis and faecalibacterium prausnitzii defines dysbiosis in patients with ulcerative colitis. Gut 63: 1275–1283.

Mahnic, A., M. Breskvar, S. Dzeroski, P. Skok, S. Pintar *et al.*, 2020 Distinct Types of Gut Microbiota Dysbiosis in Hospitalized Gastroenterological Patients Are Disease Non-related and Characterized With the Predominance of Either Enterobacteriaceae or Enterococcus. Front. Microbiol. 11: 120.

Mancabelli, L., C. Milani, G. A. Lugli, F. Turroni, D. Cocconi *et al.*, 2017 Identification of universal gut microbial biomarkers of common human intestinal diseases by meta-analysis. FEMS Microbiol. Ecol. 93: 153.

Mantilla Perez, M. B., J. Zhao, Y. Yin, J. Hu, and M. G. Salas Fernandez, 2014 Association mapping of brassinosteroid candidate genes and plant architecture in a diverse panel of Sorghum bicolor. Theor. Appl. Genet. 127: 2645–2662.

McCoy, A. N., F. Araújo-Pérez, A. Azcárate-Peril, J. J. Yeh, R. S. Sandler *et al.*, 2013 Fusobacterium Is Associated with Colorectal Adenomas. PLoS One 8: e53653.

Mikami, A., T. Ogita, F. Namai, S. Shigemori, T. Sato *et al.*, 2020 Oral administration of Flavonifractor plautii attenuates inflammatory responses in obese adipose tissue. Mol. Biol. Rep. 47: 6717–6725.

Moghimi, N., J. S. Desai, R. Bheemanahalli, S. M. Impa, A. R. Vennapusa *et al.*, 2019 New candidate loci and marker genes on chromosome 7 for improved chilling tolerance in sorghum. J. Exp. Bot. 70: 3357–3371.

Morris, G. P., P. Ramu, S. P. Deshpande, C. T. Hash, T. Shah *et al.*, 2013a Population genomic and genome-wide association studies of agroclimatic traits in sorghum. Proc. Natl. Acad. Sci. U. S. A. 110: 453–458.

Morris, G. P., D. H. Rhodes, Z. Brenton, P. Ramu, V. M. Thayil *et al.*, 2013b Dissecting Genome-Wide Association Signals for Loss-of-Function Phenotypes in Sorghum Flavonoid Pigmentation Traits. G3 Genes|Genomes|Genetics 3: 2085–2094.

Mukherjee, A., C. Lordan, R. P. Ross, and P. D. Cotter, 2020 Gut microbes from the phylogenetically diverse genus Eubacterium and their various contributions to gut health. Gut Microbes 12:.

Mukhopadhya, I., R. Hansen, C. E. Nicholl, Y. A. Alhaidan, J. M. Thomson *et al.*, 2011 A comprehensive evaluation of colonic mucosal isolates of sutterella wadsworthensis from inflammatory bowel disease. PLoS One 6: e27076.

Mural, R. V., M. Grzybowski, C. Miao, A. Damke, S. Sapkota *et al.*, 2021 Meta-analysis identifies pleiotropic loci controlling phenotypic trade-offs in sorghum. Genetics 218: 2020.10.27.355495.

Nagpal, R., S. Wang, S. Ahmadi, J. Hayes, J. Gagliano *et al.*, 2018 Human-origin probiotic cocktail increases short-chain fatty acid production via modulation of mice and human gut microbiome. Sci. Rep. 8: 1–15.

Nogal, A., P. Louca, X. Zhang, P. M. Wells, C. J. Steves *et al.*, 2021 Circulating Levels of the Short-Chain Fatty Acid Acetate Mediate the Effect of the Gut Microbiome on Visceral Fat. Front. Microbiol. 12: 1943.

Orbe-Orihuela, Y. C., E. E. Godoy-Lozano, A. Lagunas-Martínez, A. C. Castañeda-Márquez, S. Murga-Garrido *et al.*, 2022 Association of Gut Microbiota with Dietary-dependent Childhood Obesity. Arch. Med. Res. 53: 407–415.

Osaki, H., Y. Jodai, K. Koyama, T. Omori, N. Horiguchi *et al.*, 2021 Clinical response and changes in the fecal microbiota and metabolite levels after fecal microbiota transplantation in patients with inflammatory bowel disease and recurrent Clostridioides difficile infection. Fujita Med. J. 7: 87–98.

Ozato, N., S. Saito, T. Yamaguchi, M. Katashima, I. Tokuda *et al.*, 2019 Blautia genus associated with visceral fat accumulation in adults 20–76 years of age. npj Biofilms Microbiomes 5: 1–9.

Paramsothy, S., M. A. Kamm, N. O. Kaakoush, A. J. Walsh, J. van den Bogaerde *et al.*, 2017 Multidonor intensive faecal microbiota transplantation for active ulcerative colitis: a randomised placebo-controlled trial. Lancet 389: 1218–1228.

Pedret, A., R. M. Valls, L. Calderón-Pérez, E. Llauradó, J. Companys *et al.*, 2019 Effects of daily consumption of the probiotic Bifidobacterium animalis subsp. lactis CECT 8145 on anthropometric adiposity biomarkers in abdominally obese subjects: a randomized controlled trial. Int. J. Obes. 43: 1863–1868.

Queiroz, V. A. V., C. S. da Silva, C. B. de Menezes, R. E. Schaffert, F. F. M. Guimarães *et al.*, 2015 Nutritional composition of sorghum [sorghum bicolor (L.) Moench] genotypes cultivated without and with water stress. J. Cereal Sci. 65: 103–111.

Rau, M., A. Rehman, M. Dittrich, A. K. Groen, H. M. Hermanns *et al.*, 2018 Fecal SCFAs and SCFA-producing bacteria in gut microbiome of human NAFLD as a putative link to systemic T-cell activation and advanced disease. United Eur. Gastroenterol. J. 6: 1496–1507.

Rhodes, D., P. Gadgil, R. Perumal, T. Tesso, and T. J. Herald, 2017 Natural variation and genome-wide association study of antioxidants in a diverse sorghum collection. Cereal Chem. 94: 190–198.

Rhodes, D. H., L. Hoffmann, W. L. Rooney, P. Ramu, G. P. Morris *et al.*, 2014 Genome-wide association study of grain polyphenol concentrations in global sorghum [Sorghum bicolor (L.) Moench] germplasm. J. Agric. Food Chem. 62: 10916–10927.

Rios-Covian, D., S. Arboleya, A. M. Hernandez-Barranco, J. R. Alvarez-Buylla, P. Ruas-Madiedo *et al.*, 2013 Interactions between Bifidobacterium and Bacteroides species in cofermentations are affected by carbon sources, including exopolysaccharides produced by bifidobacteria. Appl. Environ. Microbiol. 79: 7518–7524.

Rowan, F., N. G. Docherty, M. Murphy, B. Murphy, J. C. Coffey *et al.*, 2010 Desulfovibrio Bacterial Species Are Increased in Ulcerative Colitis. Dis. Colon Rectum 53: 1530–1536.

Shakoor, N., G. Ziegler, B. P. Dilkes, Z. Brenton, R. Boyles *et al.*, 2016 Integration of Experiments across Diverse Environments Identifies the Genetic Determinants of Variation in Sorghum bicolor Seed Element Composition. Plant Physiol. 170: 1989–1998.

Shetty, S. A., N. P. Marathe, V. Lanjekar, D. Ranade, and Y. S. Shouche, 2013 Comparative genome analysis of Megasphaera sp. reveals niche specialization and its potential role in the human gut. PLoS One 8: e79353.

Shin, N. R., J. C. Lee, H. Y. Lee, M. S. Kim, T. W. Whon *et al.*, 2014 An increase in the Akkermansia spp. population induced by metformin treatment improves glucose homeostasis in diet-induced obese mice. Gut 63: 727–735.

So, S. Y., Q. Wu, K. S. Leung, Z. M. Kundi, T. C. Savidge *et al.*, 2021 Yeast β-glucan reduces obesity-associated Bilophila abundance and modulates bile acid metabolism in healthy and high-fat diet mouse models. Am. J. Physiol. - Gastrointest. Liver Physiol. 321: G639–G655.

Takeshita, K., S. Mizuno, Y. Mikami, T. Sujino, K. Saigusa *et al.*, 2016 A single species of clostridium Subcluster XIVa decreased in ulcerative colitis patients. Inflamm. Bowel Dis. 22: 2802–2810.

Togo, A. H., A. Diop, G. Dubourg, T. T. Nguyen, C. Andrieu *et al.*, 2016 Butyricimonas phoceensis sp. nov., a new anaerobic species isolated from the human gut microbiota of a French morbidly obese patient. New Microbes New Infect. 14: 38–48.

Ubeda, C., V. Bucci, S. Caballero, A. Djukovic, N. C. Toussaint *et al.*, 2013 Intestinal microbiota containing Barnesiella species cures vancomycin-resistant Enterococcus faecium colonization. Infect. Immun. 81: 965–973.

Ulger Toprak, N., T. Bozan, Y. Birkan, S. Isbir, and G. Soyletir, 2015 Butyricimonas virosa: The first clinical case of bacteraemia. New Microbes New Infect. 4: 7–8.

Vandenbrink, J. P., M. P. Delgado, J. R. Frederick, and F. A. Feltus, 2010 A sorghum diversity panel biofuel feedstock screen for genotypes with high hydrolysis yield potential. Ind. Crops Prod. 31: 444–448.

Wu, Y., X. Li, W. Xiang, C. Zhu, Z. Lin *et al.*, 2012 Presence of tannins in sorghum grains is conditioned by different natural alleles of Tannin1. Proc. Natl. Acad. Sci. U. S. A. 109: 10281–10286.

Yan, S., B. Yang, R. P. Ross, C. Stanton, H. Zhang *et al.*, 2020 Bifidobacterium longum subsp. longum YS108R fermented milk alleviates DSS induced colitis via anti-inflammation, mucosal barrier maintenance and gut microbiota modulation. J. Funct. Foods 73: 104153.

Yang, C., Q. Deng, J. Xu, X. Wang, C. Hu *et al.*, 2019 Sinapic acid and resveratrol alleviate oxidative stress with modulation of gut microbiota in high-fat diet-fed rats. Food Res. Int. 116: 1202–1211.

Yuan, J., C. Chen, J. Cui, J. Lu, C. Yan *et al.*, 2019 Fatty Liver Disease Caused by High-Alcohol-Producing Klebsiella pneumoniae. Cell Metab. 30: 675-688.e7.

Zhan, Z., W. Liu, L. Pan, Y. Bao, Z. Yan *et al.*, 2022 Overabundance of Veillonella parvula promotes intestinal inflammation by activating macrophages via LPS-TLR4 pathway. Cell Death Discov. 8: 1–12.

Zhang, D., J. Li, R. O. Compton, J. Robertson, V. H. Goff *et al.*, 2015 Comparative Genetics of seed size traits in divergent cereal lineages represented by sorghum (Panicoidae) and Rice (Oryzoidae). G3 Genes, Genomes, Genet. 5: 1117–1128.

Zhang, X., Z. Ning, J. Mayne, Y. Yang, S. A. Deeke *et al.*, 2020 Widespread protein lysine acetylation in gut microbiome and its alterations in patients with Crohn’s disease. Nat. Commun.

Zhao, J., M. B. M. Perez, J. Hu, and M. G. S. Fernandez, 2016 Genome-Wide Association Study for Nine Plant Architecture Traits in Sorghum. Plant Genome 9: plantgenome2015.06.0044.

Zheng, Z., S. Hey, T. Jubery, H. Liu, Y. Yang *et al.*, 2020 Shared Genetic Control of Root System Architecture between Zea mays and Sorghum bicolor. Plant Physiol. 182: 977–991.

Zhou, Y., S. Srinivasan, S. V. Mirnezami, A. Kusmec, Q. Fu *et al.*, 2019 Semiautomated Feature Extraction from RGB Images for Sorghum Panicle Architecture GWAS. Plant Physiol. 179: 24–37.
