# Supplementary material for: Genomic co-localization of variation affecting agronomic and human gut microbiome traits in a meta-analysis of diverse sorghum
Source: G3 (Bethesda). 2024 Jul 9;14(9):jkae145. doi: 10.1093/g3journal/jkae145 (PMC11373648; doi:10.1093/g3journal/jkae145)
Supplement: jkae145_Supplementary_Data [file jkae145_supplementary_data.zip › Supplemental_Material_Legends_G3-2024-405129.docx]

**Supplemental Materials for**

**Genomic co-localization of variation affecting agronomic and human gut microbiome traits in a meta-analysis of diverse sorghum**

Nate Korth *et al.*

*Corresponding author. Email: [abenson1@unl.edu](mailto:abenson1@unl.edu)

**Supplementary Figures**

Figure S1. Correlation of the prebiotic index to butyrate.

Figure S2. Genus level abundance of microbiomes from two subjects used in mapping experiment.

Figure S3. LD analysis and linkage type of all associated markers in each MEL. Each column shown indicates a single marker associated with one or more microbial taxa. Cells are colored based on linkage ($R^{2}$ value).

Figure S4. Beta-diversity analysis of MEL6A validation. Beta-diversity (Weighted Jaccard index) analysis of 12 human microbiomes screened with sorghum lines pooled by allele at MEL6A and tannin content. Samples are colored based on MEL6A haplotype and shaped based on tannin content. P-values shown are a result of a PERMANOVA test for significance between Haplotype groups.

Figure S5. Selected GWAS output. GWAS output for *Faecalibacterium* at ASV and genus level, the latent variable and principal component with the most total significantly associated markers, and Prebiotic Index for both subjects and Five seed composition and biochemical phenotypes demonstrating similar patterns of associations as microbiome metrics.

**Supplementary Tables**

Table S1. List of sorghum genotypes used in study.

Table S2. Description of agronomic traits used in study.

Table S3. Microbial genera that comprise the "Prebiotic Potential Index".

Table S4. Heritability values for microbiome genera calculated in small scale experiment with microbiomes from eight human subjects.

Table S5. Heritability values for microbiome genera calculated in large-scale mapping experiment.

Table S6. Description of LD calculations for candidate loci.

Table S7. Description of all MEL identified by RMIP GWAS.

Table S8. Results of ANOVA analysis from Faeclaibacterium qPCR data from validation study

**Supplementary datasets:**

Data S1. BLUES calculated for all microbiome metrics and sorghum agronomic traits.

Data S2. Compiled GWAS output from all traits.

Data S3. Faecalibacterium qPCR data from Validation study
