# Supplementary figures and images for: Genomic co-localization of variation affecting agronomic and human gut microbiome traits in a meta-analysis of diverse sorghum
Source: G3 (Bethesda). 2024 Jul 9;14(9):jkae145. doi: 10.1093/g3journal/jkae145 (PMC11373648; doi:10.1093/g3journal/jkae145)

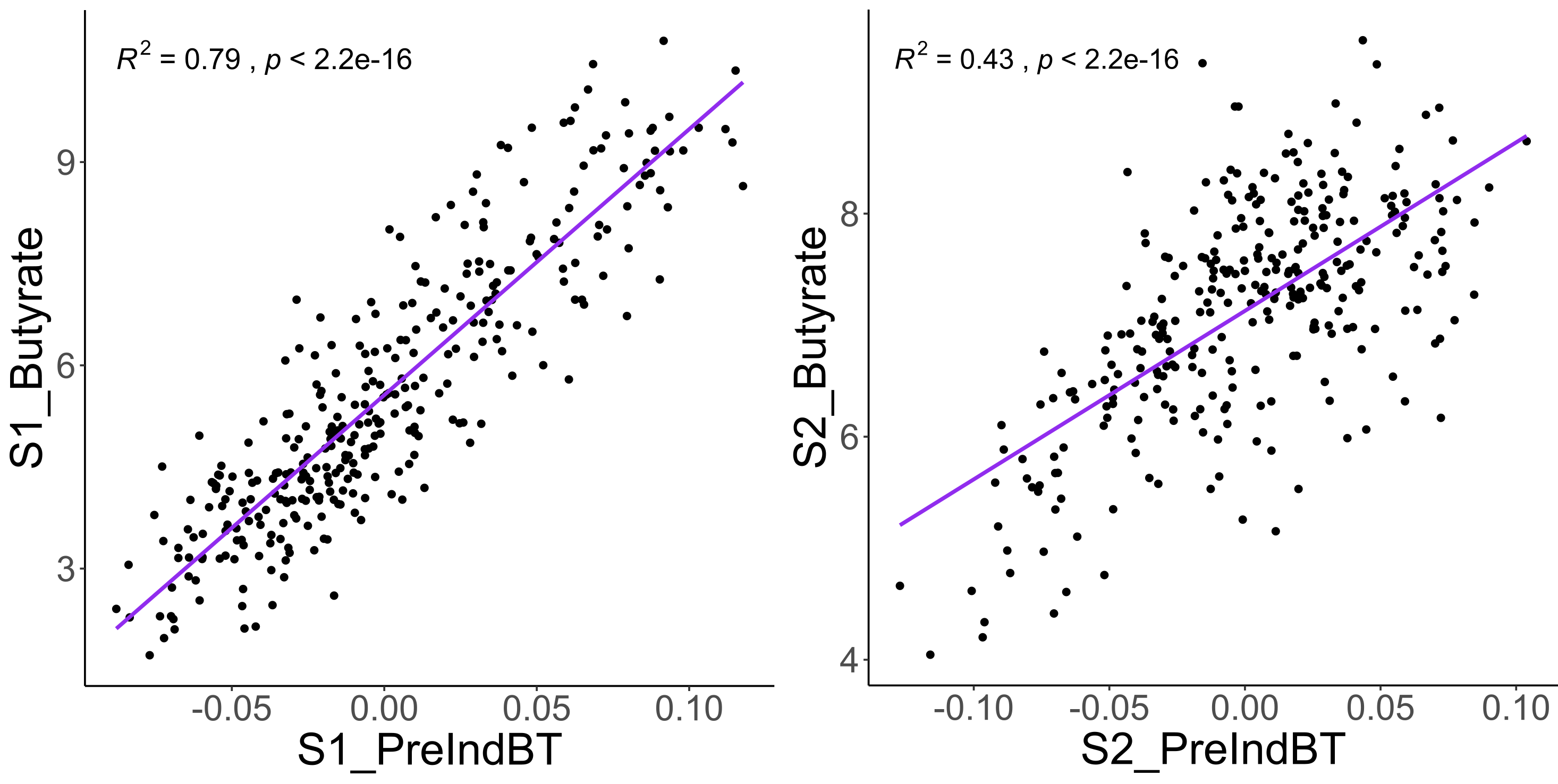

Supplement: jkae145_Supplementary_Data [file jkae145_supplementary_data.zip › Figure_S1_G3-2024-405129.tif]

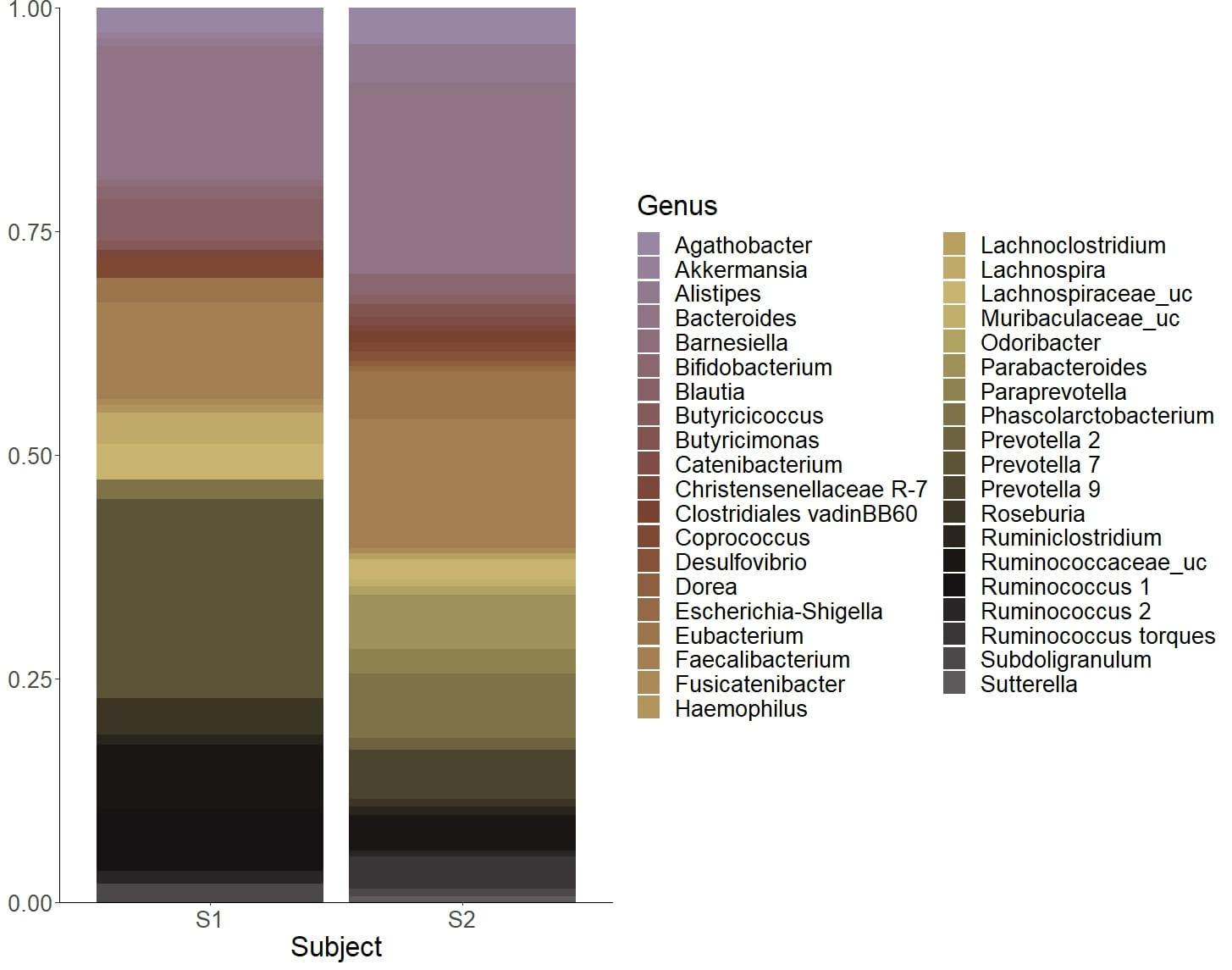

Supplement: jkae145_Supplementary_Data [file jkae145_supplementary_data.zip › Figure_S2_G3-2024-405129.tif]

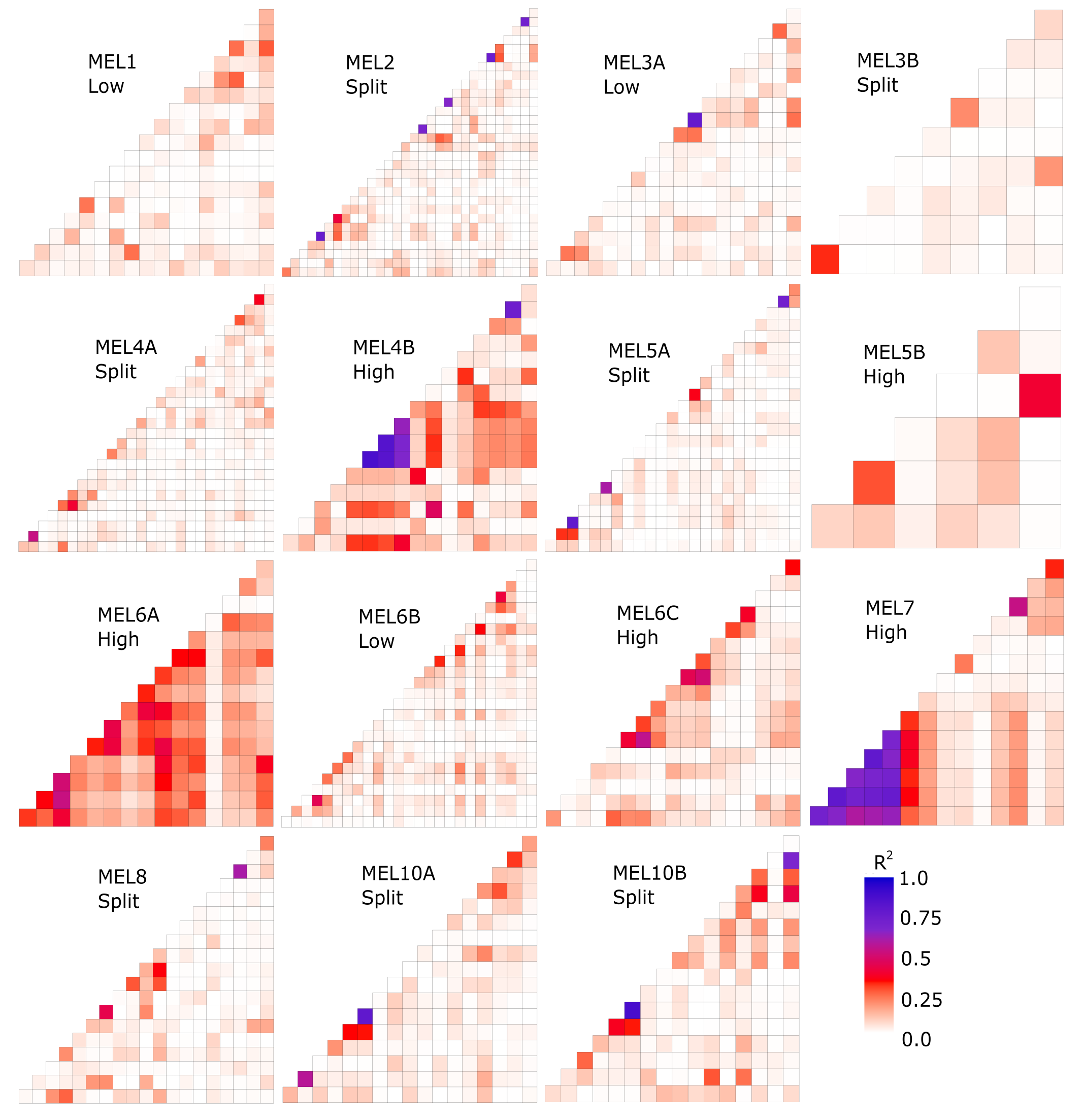

Supplement: jkae145_Supplementary_Data [file jkae145_supplementary_data.zip › Figure_S3_G3-2024-405129.tif]

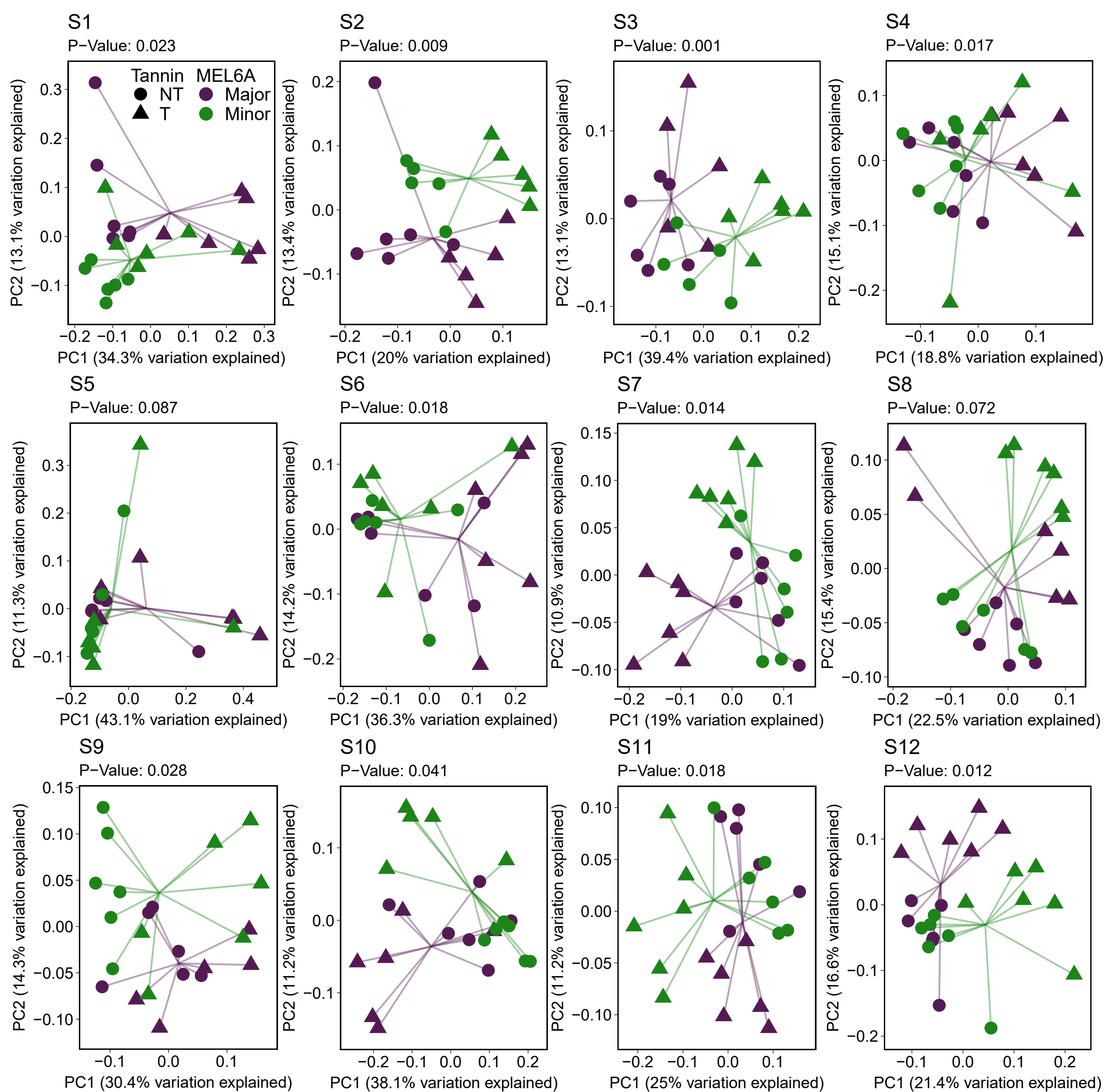

Supplement: jkae145_Supplementary_Data [file jkae145_supplementary_data.zip › Figure_S4_G3-2024-405129.pdf]

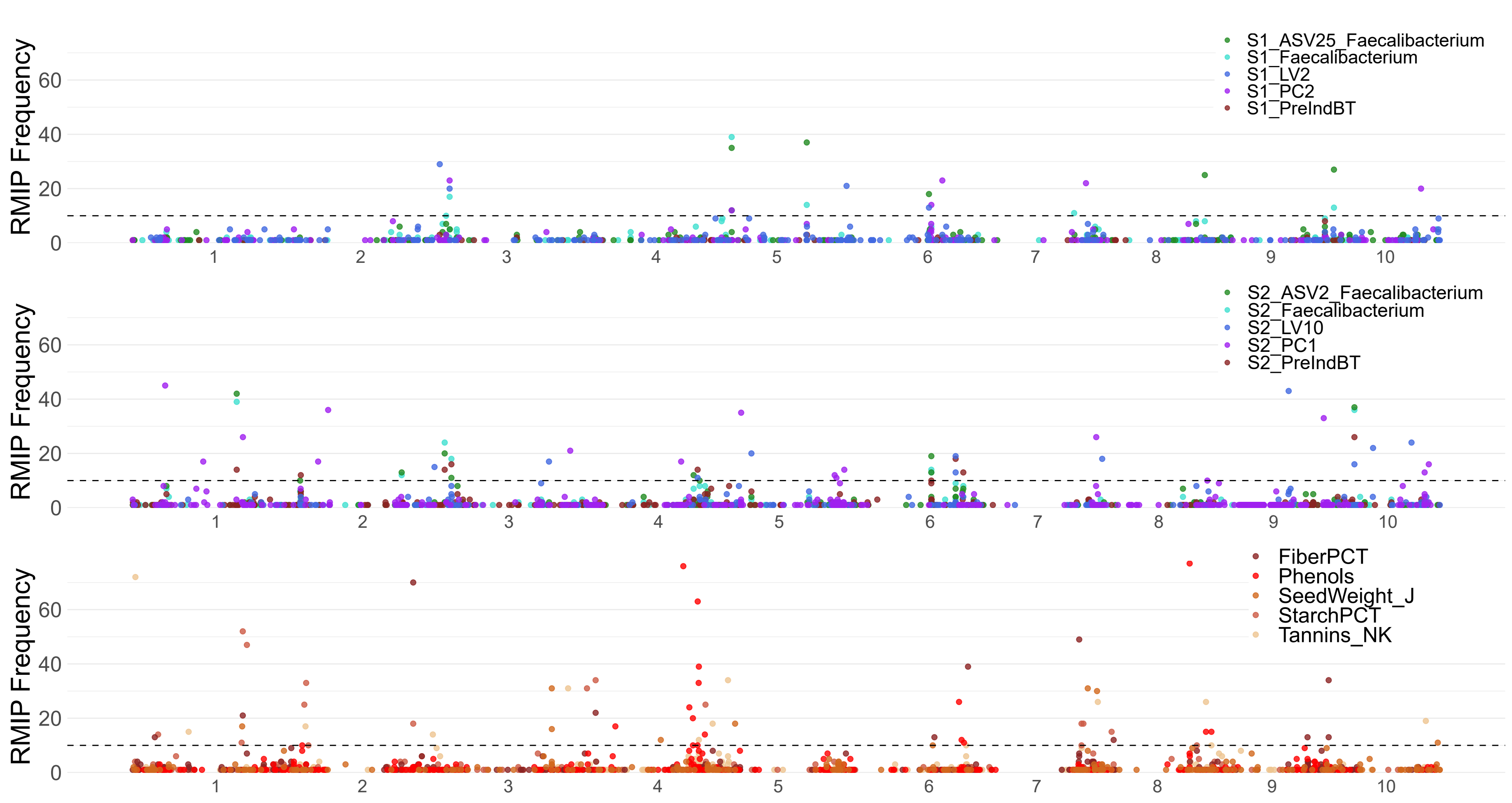

Supplement: jkae145_Supplementary_Data [file jkae145_supplementary_data.zip › Figure_S5_G3-2024-405129.pdf]
